# Body image disturbance, interoceptive sensibility and the body schema across female adulthood: a pre-registered study

**DOI:** 10.3389/fpsyg.2023.1285216

**Published:** 2023-11-30

**Authors:** Akansha M. Naraindas, Sarah M. Cooney

**Affiliations:** School of Psychology, University College Dublin, Dublin, Ireland

**Keywords:** body image disturbance, motor imagery, ageing, interoceptive sensibility, mental rotation

## Abstract

**Introduction:**

Body image disturbance (BID) typically involves explicit negative attitudes toward one’s shape and weight and is associated with altered interoceptive sensibility (the subjective perception of internal bodily states). This association is a known risk factor for the development and maintenance of eating disorders. However, while research has centred on younger women with eating disorders, diverse facets of BID appear in women without eating disorders across adulthood. Research shows that in the general population, young women (ages 18–25) with high BID exhibit disturbances in the body schema: an implicit sensorimotor representation of the body in space which includes mental simulation of a movement such as motor imagery. Given that body image is subject to age-related influences, it is important to investigate how age-related variation in BID can influence the body schema beyond young adulthood alone. Here, we examine the relationship between BID, interoceptive sensibility and the body schema across female adulthood.

**Methods:**

Cross-sectional data was collected online from 1,214 women across four age groups: Young adults (18–24), Adults (25–39), Middle-aged adults (40–59), and Older aged adults (60–75). BID was indexed by questionnaires measuring body objectification, state, and trait body dissatisfaction. Interoceptive sensibility (IS) was measured using the MAIA-2 questionnaire. The body schema was evaluated through the Own Body Transformation task: a mental rotation task which assesses the capacity to make an embodied mental transformation.

**Results:**

Analyses revealed that while body objectification and trait body dissatisfaction decreased from young to older adulthood, state body dissatisfaction showed a marked increase. A negative relationship between IS and BID across all age groups was also evidenced. Finally, age, BID and orientation of the presented body were significant predictors of the time taken to make an embodied transformation.

**Discussion:**

These findings highlight the consistent relationship of BID and IS across age groups beyond young adulthood and demonstrate the varying importance of different aspects of BID as individuals age. We also evidence for the first time that disruptions in body image have the potential to impact implicit sensorimotor representations of the body even in women without eating disorders across female adulthood.

## 1 Introduction

Body image disturbance (BID) is a multidimensional concept encompassing perceptual, behavioural, and cognitive distortions associated with weight or shape (Cash and Deagle, 1997). The disturbance typically occurs in relation to explicit negative attitudes and evaluations of one’s body, known as body image (Cash and Deagle, 1997; Skrzypek et al., 2001). Individuals with high levels of BID, as seen in eating disorders (EDs), also show disruptions in the body schema—an implicit sensorimotor representation of the body’s positioning in space and action (de Vignemont, 2010). As such, individuals with EDs perceive their body as larger than its actual size and simultaneously interact with their body as if it occupies a greater space during action (Guardia et al., 2010, 2012; Beckmann et al., 2021). To date links between BID and body schema have primarily been researched in the context of young women diagnosed with EDs (Guardia et al., 2010; Keizer and Engel, 2021; Meregalli et al., 2022). However, the detrimental impacts of negative body image persist in women across the lifespan, extending beyond young adulthood (Stice and Shaw, 2002; Kilpela et al., 2015). Despite this, the developmental variations, and relationships between attitudinal and the sensorimotor components of BIDs across the lifespan of women remain unknown. Emerging evidence suggests that interoception, the awareness of the internal bodily self (Craig, 2002), has a notable influence on both body image (Badoud and Tsakiris, 2017) and recently the body schema (Baumann et al., 2022). Therefore, this study seeks to explore, for the first time, the relationship between BID, interoception and body schema across distinct epochs of female adulthood.

Body dissatisfaction is a significant component of BID and comprises negative thoughts and emotions related to the physical appearance of one’s body including body size, weight, shape, and attractiveness (Cash et al., 2004). Body dissatisfaction typically arises during adolescence (Wang et al., 2019), and remains relatively consistent into adulthood (19–40 years) (Runfola et al., 2013). However, research is less consistent about body dissatisfaction in middle-late adulthood (40–75 years) where some studies indicate that body dissatisfaction and appearance investment decrease (Runfola et al., 2013; Kilpela et al., 2015) and other studies report body dissatisfaction increases (Gagne et al., 2012). Although research into negative body image among women has largely concentrated on a narrow age bracket of university undergraduates aged 18–24 (Tiggemann, 2004; Slevec and Tiggemann, 2011), existing studies show that body dissatisfaction can have enduring negative effects on women throughout their lives (Cash et al., 2004; Tiggemann, 2004; Mangweth-Matzek et al., 2006; Mond et al., 2013).

However, body dissatisfaction alone is not sufficient to fully explain the range of experiences related to negative body image across adulthood (Tiggemann and Lynch, 2001). Firstly, body dissatisfaction can be experienced as either an immediate state or a stable trait across time (Cash et al., 2002). Understanding how transient state body dissatisfaction relates to the formation of lasting trait body dissatisfaction is crucial in comprehending the progression of BID (Leahey and Crowther, 2008; Colautti et al., 2011). Secondly, other components of negative body image, such as self-objectification, are believed to have varying effects on body dissatisfaction across different stages of adulthood (Tiggemann and Lynch, 2001). Self-objectification involves engaging in body surveillance, which entails viewing and evaluating one’s body as an object to conform to societal expectations and to avoid body shame (McKinley and Hyde, 1996). Body surveillance is a common risk factor for BIDs (Lindner et al., 2012; Jackson and Chen, 2015) and even eating pathology in women across the lifespan (Tiggemann and Lynch, 2001; Tylka, 2004; Grabe et al., 2007; Slater and Tiggemann, 2015). Younger women are considered to be more susceptible to societal influence and body ideals, demonstrating higher levels of body surveillance and body shame compared to older women (Tiggemann and Lynch, 2001; Grippo and Hill, 2008). However, women undergo pivotal life transitions throughout adult life, such pregnancy and menopause, characterized by physiological changes that can cause deviations from societal body ideals (Erbil, 2018). In mid-life, women undergo significant hormonal shifts, especially during the menopausal period, leading to decreased levels of estrogen and progesterone (Soares and Zitek, 2008). These hormonal fluctuations influence mood, behavior, and also body composition, resulting in the redistribution of body fat, decreased muscle mass, and changes in skin firmness and elasticity (Soares and Zitek, 2008; St-Onge and Gallagher, 2010). Coupled with immediate life stressors such as caregiving responsibilities, work demands, and household obligations, this can limit the time available for self-care and grooming practices (McLean et al., 2010; Kilpela et al., 2015). As a result, women in middle to older adulthood also experience high levels of negative body image albeit, across different domains of BID compared to younger women (Roy and Payette, 2012; Carrard et al., 2021). Therefore, there is a need for a comprehensive exploration of the distinct characteristics of the occurrence of negative body image across different stages of life, particularly in mid to older adulthood.

While body image involves more explicit aspects of body representation, the body schema is an implicit sensorimotor representation of the body that is engaged during movement (Head and Holmes, 1911; Gallagher, 2005; de Vignemont, 2010). According to the embodiment approach, the body schema also plays a role in action planning via mental simulation of action, i.e., motor imagery (Jeannerod, 2001). In motor imagery tasks, individuals mentally rotate their own body to match a displayed body on a screen (Schwoebel et al., 2001; Coslett et al., 2010). This process, known as egocentric mental rotation (Zacks et al., 2000; Kaltner et al., 2014), relies on one’s body schema as a foundational basis for the judgement. There is now mounting evidence that those with clinical BIDs (e.g., individuals with EDs) show a disturbance in the egocentric reference frame during body-based mental rotation (Urgesi et al., 2011; Cipolletta et al., 2017; Meregalli et al., 2022). Studies assessing whole-body mental rotations in individuals with BIDs typically use variations of the Own Body Transformation task (OBT) (Blanke et al., 2005; Gardner et al., 2012) which involve making speeded left- and right-hand judgments of full-bodied avatars in either front-facing or back-facing positions (Urgesi et al., 2011; Cooper and Mohr, 2012; Cipolletta et al., 2017). Individuals with EDs demonstrate altered reaction times and accuracy in the OBT task compared to non-ED controls, indicating disturbances in egocentric processing in ED individuals (Urgesi et al., 2011; Cooper and Mohr, 2012; Serino et al., 2015; Cipolletta et al., 2017; Meregalli et al., 2022). However, the evidence remains equivocal regarding the relationship between BIDs, altered egocentric processing, and their effect on the body schema in individuals without EDs. Some studies found a relationship between negative body attitudes and altered body schema among healthy women in body-scaled action, e.g., estimating passage through a doorway (Guardia et al., 2010; Keizer et al., 2013; Irvine et al., 2019), while others have not (Wignall et al., 2017; Glashouwer et al., 2019). Furthermore, research indicates that ageing causes declines in the body schema and the bodies spatial representations in motor imagery tasks (See: Costello and Bloesch, 2017 for review). These inconsistencies highlight the need for further investigation of the aetiology of BID and its relationship to the body schema in non-clinical populations across age.

One explanation that links altered body schema function to BID is the Allocentric Lock Theory (ALT). The ALT suggests that BID arises from impairments in egocentric processing, resulting in an inability to update the allocentric mental representation of the body schema in memory (Riva, 2014). This disruption is speculated to occur due to exogenous stressors like negative body image (e.g., body dissatisfaction, body objectification) which may affect how internal body-related sensory information (e.g., interoception) is processed (Riva and Dakanalis, 2018). Interoception involves the awareness of and attention toward internal physiological signals representing the body’s condition and contributes to the egocentric body experience (Craig, 2002; Tsakiris et al., 2011). As a multidimensional concept, interoception spans conscious and unconscious levels (refer to Garfinkel et al., 2015 for a review). Interoceptive sensibility (IS) pertains to self-reported awareness of internal sensations (Khalsa et al., 2018), and has demonstrated associations with body image in individuals both with and without EDs (Eshkevari et al., 2014; Jenkinson et al., 2018). Studies with non-ED participants show a consistent inverse relationship between IS, and negative body image measures (e.g., body dissatisfaction, self-objectification) (Myers and Crowther, 2008; Ainley and Tsakiris, 2013; Emanuelsen et al., 2015; Todd et al., 2019a, 2019b). Moreover, studies examining the body schema have shown that different aspects of interoceptive processing are linked to performance in motor imagery, such as in the OBT task (Heydrich et al., 2021) and in body-scaled action tasks (Baumann et al., 2022). Taken together, these findings point toward interoception playing a crucial role in building and maintaining image and schema-based body representations.

However, the interactions between body image and interoception are complex as both concepts are multidimensional (Khalsa et al., 2018; Prnjak et al., 2022) and are independently influenced by individual differences, especially age e.g., body image: (Tiggemann, 2004; Karazsia et al., 2017; Hockey et al., 2021; Lacroix et al., 2023), interoception: (Murphy et al., 2018; Raimo et al., 2021). As such, it is unclear how both body image and interoception interact with the body schema within the context of influential sociodemographic characteristics such as age. Therefore, the objective of this study is to examine the impact of age-related differences in body image and interoceptive sensibility on performance in a task that indexes egocentric mental transformation. The study adopts a cross-sectional design, recruiting adult females across a range of age groups, spanning from young to older adulthood. To assess dimensions of BID, including trait and state body dissatisfaction and self-objectification, self-report questionnaires are used. IS is also appraised through a self-report questionnaire. Additionally, egocentric mental transformation is assessed using a modified version of the OBT task (Zacks et al., 1999; Blanke et al., 2005).

As such, based on the discussed literature we hypothesize the following:

1. Regarding OBT task performance:
  - The egocentric transformation cost will differ according to the orientation (0, 90) of the body presented. There will be larger egocentric transformation costs at 90 degrees compared to 0 degrees. We expect smaller egocentric transformation costs for back facing to-side facing avatars.
  - We expect significant differences in egocentric transformation costs between the age groups. We predict that the egocentric transformation cost will decrease with age (Costello and Bloesch, 2017).
  - We also manipulate avatar weight in the modified OBT task. Accordingly, we anticipate variations in task performance based on the weight of avatars. Specifically, we predict larger egocentric transformation costs for underweight avatars compared to overweight avatars.
2. Regarding Interoceptive sensibility, we expect that IS will decrease with age in line with finding of Khalsa et al. (2009).
3. Regarding BID and its individual dimensions, including trait and state body dissatisfaction and self-objectification, we predict that all scores will exhibit a decline with age, aligning with the findings of Grippo and Hill (2008).
4. We expect that BID will be predicted by interoceptive sensibility and age (Tiggemann, 2004; Ainley and Tsakiris, 2013).
5. We expect that the egocentric transformation cost will be predicted by IS and BID across age (Urgesi et al., 2011; Irvine et al., 2019; Raimo et al., 2021).

## 2 Materials and methods

### 2.1 Participants

The study recruited female participants from four different age groups: *Young Adults* (18–24 years old), *Adults* (25–39 years old), *Middle-aged Adults* (40–59 years old) and *Older Adults* (60–75 years old) as classified by the age standards put forward by the World Health Organisation (United Nations, 2015). Participants were screened based on whether they identify as female, they are right-handed/ ambidextrous, have normal or corrected-to-normal visual acuity, their ability to read and write in English, and the absence of neurological disorders and a Prolific approval rate greater than 90%. Participants were recruited online through the research participant crowdsourcing platform Prolific^1^ and were paid £8.50/h for the duration of the study. The formula used to calculate the minimum number of participants needed for the regression was n = 100 + 50i, where i refers to the number of independent variables in the final model (Bujang et al., 2018). In the most complex regression model, the maximum number of predictors not including age group is 4. Therefore, a minimum sample size of 300 participants per age group (total = 1,200) would be sufficient to derive the statistics that represent the behavioural parameters. The total sample consisted of 1,214 female participants. With 301 in the *Young Adult* group (Mean Age = 21.60, *SD* = 1.96, range = 18–24), 300 in the *Adult* group (Mean Age = 29.16, S*D* = 3.87, range = 25–39), 298 in the *Middle Adult* group (Mean Age = 48.22, *SD* = 5.63, range = 40–59), and 314 in the *Older Adult* group (Mean Age = 64.61, *SD* = 3.93, range = 60–75).

We did not exclude participants based on ethnicity; instead, we accounted for ethnicity as a demographic factor (See Supplementary file S7 for full breakdown of participants ethnicity). A majority of the participants (82%) in this study come from countries with a high human development index ≥0.70 (e.g., United Kingdom, United States, Portugal, Poland, Spain, Greece, Canada, Germany, and South Africa) indicating high levels of socioeconomic development (United Nations, 2019). Considering that research suggests a link between body image concerns and Westernization, urbanization, and economic progress (Becker, 2004; McLaren and Kuh, 2004; Gorrell et al., 2019), we expect that these countries may exhibit similar levels of body image concerns.

### 2.2 Materials

This was a cross-sectional pre-registered^2^ online study involving a within-subjects repeated measures design with three measures: Body image disturbance, measured by questionnaires: Body Shape Questionnaire (Cooper et al., 1987), Body Image State Scale (BISS) (Cash et al., 2002), Objectified Body Consciousness (OBC) questionnaire (McKinley and Hyde, 1996), a measure of interoceptive sensibility (MAIA-2) (Mehling et al., 2018) and a modified version of the OBT task. The study was presented online on Qualtrics (Qualtrics, Provo, UT) and Psychopy (version 2021.1.3; (Peirce, 2009)) via the Pavlovia platform.^3^

### 2.3 Questionnaires

#### 2.3.1 Body image disturbance

To construct a composite of BID and investigate its relationship to the body schema we use the construct of body image disturbance derived from the Allocentric Lock Theory (Riva, 2014) as mentioned in the introduction. Wherein, body image disturbance includes self-surveillance, body shame and body dissatisfaction.

##### 2.3.1.1 Body dissatisfaction

###### 2.3.1.1.1 Trait body dissatisfaction

Trait body dissatisfaction was measured with the Body Shape Questionnaire (BSQ) (Cooper et al., 1987) which contains 30 questions. Four questions were omitted as they were about weight control/eating disorder behaviours and were not necessary for our study. The BSQ has good concurrent and discriminative validity (Cooper et al., 1987) and has been tested in clinical and ED samples (Probst et al., 2008) as well as in the general population (Franko et al., 2012). Moreover, the BSQ has been validated in 18–50 (Wade, 2016) and 50-75-year-olds (Sánchez-Cabrero et al., 2020).

###### 2.3.1.1.2 State body dissatisfaction

State body dissatisfaction was measured using the Body Image State Scale (BISS) (Cash et al., 2002) which measures state (current) levels of body dissatisfaction. Participants were shown six statements about body dissatisfaction and were asked to pick statements from a 9-point Likert scale based on how they felt at that very moment. Scores on the BISS were reverse coded so that higher scores indicated higher levels of BID. The construct validity of BISS has been established through experiments with varying reactions to appearance-related information based on the degree of dysfunctional body image investment (Cronbach’s *α* > 0.87 for all versions) (Cash et al., 2002).

##### 2.3.1.2 Body objectification

###### 2.3.1.2.1 Body shame

Body shame was measured using the shame subscale of the Objectified Body Consciousness (OBCS) questionnaire (McKinley and Hyde, 1996). Containing eight questions, scored on a 7-point Likert scale from “strongly agree” to “strongly disagree” the body shame sub-scale has high internal consistencies *a* = 0.75 (McKinley and Hyde, 1996), *a* = 0.78 (Greenleaf and McGreer, 2006) and *a* = 0.79 (Forbes et al., 2006). Concurrent validity is supported by significant positive correlations between the Body shame scale and measures of individuals with disordered eating (*ß* = 0.398, *p* = 0.007) (Greenleaf and McGreer, 2006).

###### 2.3.1.2.2 Body surveillance

Body surveillance was measured by the body surveillance subscales of the OBCS (McKinley and Hyde, 1996). This scale contains eight questions each, scored on a 7-point Likert scale from “strongly agree” to “strongly disagree.” Participants were given a total score out of 112 with higher scores reflecting higher levels of body surveillance. The body surveillance subscale has a Cronbach’s α of 0.84 (McKinley and Hyde, 1996).

#### 2.3.2 Interoceptive sensibility

Measured by the Multidimensional Assessment of Interoceptive Awareness (MAIA-2) (Mehling et al., 2018) the scale contains 37 items, scored on a 5-point Likert scale from “Never” to “Always.” The scale contains 37 items, scored on a 5-point likert scale from “Never” to “Always.” The MAIA-2 consists of 8 sub-scales including: Noticing, Not-distracting, Not-Worrying, Attention Regulation, Emotional Awareness, Self-Regulation, Body Listening, Trust (See: Mehling et al., 2018, for review on sub-scales). Subscale scores are calculated by summing responses and dividing by the number of items in each subscale. However, as we wanted an overall index of IS, we calculated an overall total by summing all the items together. While the utilization of a total score is not usually advised for the MAIA-2 (Mehling et al., 2018), we sought to capture an overall index of IS to include in a regression model. Participants were given a total score of 185 with higher scores reflecting higher levels of interoceptive sensibility. The 8-factor model of the MAIA-2 was confirmed with appropriate fit indices [RMSEA = 0.055 (95% CI 0.052–0.058); SRMR = 0.064] and improved internal consistency reliability (Mehling et al., 2018).

#### 2.3.3 Own body transformation task

**Figure 1:**
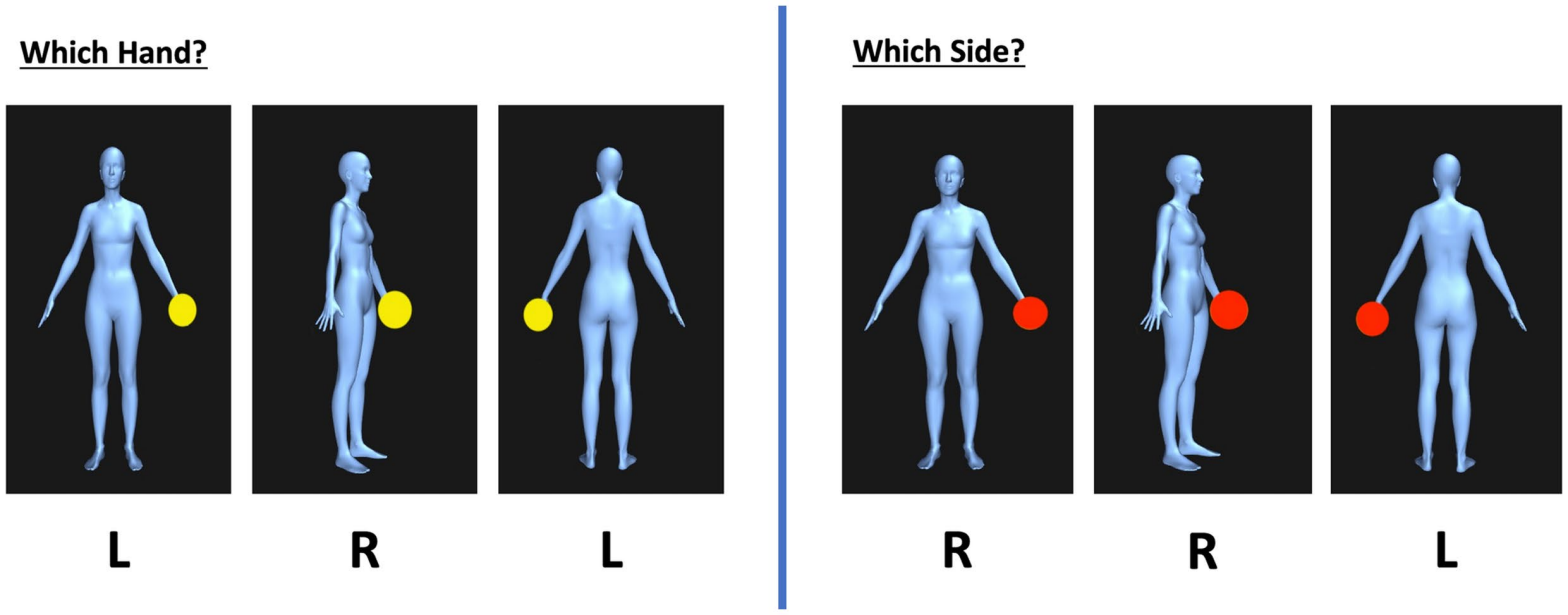
Example of the avatars from the OBT task as created on http://bodyvisualizer.com (Perceiving Systems MPI IS, 2011) with accurate laterality judgments shown below the images in the which hand condition (experimental) and the which side condition (control).

Constructed on https://bodyvisualizer.com/, (Perceiving Systems MPI IS, 2011) the stimuli were avatars of full-body female dimorphic figures with either a red or yellow ball placed on the left or right hand (see Figure 1). The avatars were displayed at angular disparities of 0 and 90-degree orientations, and presented in three positions: front-facing, back-facing, and side-facing. The accuracy and speed of laterality judgments in this task are typically enhanced when the avatars’ body is presented in positions and orientations that are most closely aligned with the observer’s own body, i.e., the 0-degree back-facing position (Parsons, 1987; Sirigu et al., 1996). Any variations in response to the orientations of the stimuli can be attributed to the influence of biomechanical constraints on the participant’s response profile (Ionta et al., 2012). To vary the size of the stimuli the bodies of the avatars were made to appear marginally over and marginally under the average BMI of 22 by +/− 5 BMI points (e.g., underweight = 17 BMI, overweight = 27 BMI). Variations in size and position were used to prevent participants from associating specific stimulus types with particular laterality judgments in the task (Zacks et al., 1999).

In the experimental trials, the avatars had a yellow ball in their hand, and participants were instructed to imagine that the avatar’s body was their body and that they were in the same position as the avatar. Participants were required to make a 2AFC judgement regarding what hand the ball was in: their left hand or their right hand as seen in Figure 1. They were instructed to respond as quickly as possible whilst continuing to respond as accurately as possible by pressing the left or right arrow key on the keyboard. In this task, we predicted that participants would take longer to mentally rotate front-facing and side-facing avatars, as they were contralateral to the participant’s actual body position, compared to back-facing avatars which were ipsilateral to the participant’s own body position.

During control trials, a red ball was presented to signal a different task requirement. Participants were instructed to determine the side of the screen where the ball appeared, rather than focusing on which hand the ball was associated with (as in experimental trials), as depicted in Figure 1. The control trials did not require participants to use motor imagery to make an embodied transformation, thus the time taken to make “which side” judgments enabled us to ascertain the cost to the response time of making an embodied transformation (Gardner et al., 2012). Control rials were displayed in 0 degrees only, to be compared with 0-degree experimental trials. Internal validity and reliability of OBT tasks are shown across multiple different studies in clinical and healthy populations (Gardner et al., 2012; Gronholm et al., 2012).

### 2.4 Procedure

This was an online study where participants were instructed to complete the tasks on a laptop or computer and to have the browser in full-screen mode. The order of the tasks was counterbalanced within each age group, with half of the participants completing the OBT task first followed by the questionnaires and the other half completing the questionnaires first followed by the OBT task.

In the OBT task participants were given instructions on how to perform the tasks and then given 10 practice trials. There were four blocks of 36 trials, of which 24 were experimental trials and 12 were control trials, resulting in 96 trials in total. For the experimental trials, stimuli were presented once per stimulus combination: *orientation* (0, 90), *position* (front-facing, back-facing, side-facing), and *weight* (below average BMI, above average BMI). This was a forced choice task and key press responses that corresponded with the direction of judgement were required to proceed to the next trial and response times were recorded accordingly. Additionally, three attention check trials were randomly presented to ensure participants were paying attention. The total time taken to complete the task was approximately 10–15 min.

Participants were directed to a survey where demographic data: participants’ age, ethnicity, presence of existing mental health conditions and whether they had/have an eating disorder were recorded. This was followed by the BSQ (Cooper et al., 1987), BISS and the Body Shame and Body Surveillance subscales of the OBCS (McKinley and Hyde, 1996) in that order. Finally, participants were given the MAIA-2 (Mehling et al., 2018) to assess interoceptive sensibility.

## 3 Results

### 3.1 Data analysis

First, descriptive statistics were computed for all variables included in the study. The BID composite score was calculated as a combination of the scores on the four body image.

questionnaires (BSQ, BISS and body shame and body surveillance subscales of the OBCS). The scores on these questionnaires were z-standardised to allow for cross-scale comparison. The z scores represent differences in standard deviation units, (i.e., the mean at each time point minus the grand mean of all the observations divided by the overall standard deviation). The BID composite was calculated by adding the z-scores of all the questionnaire measures divided by the number of questionnaires (Andrade, 2021). The raw means and standard deviations of the scores for each questionnaire are reported by age group as seen in Table 1. Effect sizes, (Cohens ‘d for t-tests and partial eta squared for ANOVA’s) were used to estimate the main effects and between-group differences. Greenhouse–Geisser corrections were used when Mauchly’s test for sphericity was significant and Bonferroni corrections for multiple comparisons were applied to *post hoc* tests where appropriate.

**Table 1:** Mean BID *z* composite scores and mean raw scores for the BISS, BSQ, Surveillance, Shame, Interoceptive sensibility (MAIA-2) and split by age group (Means and SDs).

Questionnaires	Young adults (*N* = 301)	Adults (*N* = 300)	Middle adults (*N* = 298)	Older adults (*N* = 314)
BID (*z* composite)	0.252 (2.62)	0.225 (3.01)	0.107 (2.64)	0.594 (2.57)
BSQ	88.61 (33.43)	89.79 (36.22)	86.73 (30.76)	79.26 (29.02)
BISS	30.34 (9.62)	31.16 (10.33)	33.78 (9.51)	32.92 (9.24)
Body Surveillance	35.80 (8.32)	34.38 (9.11)	31.99 (8.86)	28.96 (8.87)
Body Shame	28.51 (8.59)	28.90 (9.89)	27.69 (8.78)	25.76 (8.04)
Interoception (MAIA-2)	118.65 (15.89)	120.16 (16.38)	117.61 (17.53)	115.29 (15.25)

For the OBT task, participants failing more than two attention checks in the task were not included in the analysis (*N* = 8). Response latencies faster than 200 ms and slower than 5,000 ms were removed from the analysis (Harris et al., 2012). Additionally, to identify RT outliers, a within-participant threshold was calculated using the mean of each participant on each position condition (back-facing, front-facing, and side-facing). The reaction time outliers were.

defined as 3.5 standard deviations from the individual participant RT mean for that condition; with overall 377 trials removed (0.38%). Mean RTs on correct trials are reported for Position (back-facing, front-facing, and side-facing trials) and Orientation (0, 90) in Table 2. An egocentric transformation index was calculated by subtracting the mean RT on correct trials for back-facing trials from front-facing trials (Thakkar et al., 2009). This transformation index was subsequently used as the outcome variable in the regression analysis. Statistical analyses were conducted using R Statistics (R Core Team, 2021) and JASP (Team, JASP, 2023).

**Table 2:** The Mean (SD) reaction times (RTs) on correct trials (ms) on the own body transformation (OBT) task in each age group for orientation and position and Egocentric transformation.

Age group	Back-facing		Front-facing		Egocentric transformation (front-facing - back-facing)	
	0 degrees	90 degrees	0 degrees	90 degrees	0 degrees	90 degrees
Young Adults	1254.201 (426.42)	1438.061 (524.81)	1774.314 (600.66)	1689.223 (571.67)	517.812 (385.472)	250.461 (330.517)
Adults	1367.783 (522.17)	1551.589 (621.82)	1886.987 (656.60)	1803.062 (652.56)	512.501 (380.371)	241.856 (356.268)
Middle Adults	1418.732 (456.10)	1540.181 (538.16)	1866.371 (582.20)	1801.347 (601.01)	444.022 (376.018)	257.526 (338.914)
Older Adults	1651.261 (478.87)	1787.07 (540.1)	2095.255 (615.17)	2015.246 (576.85)	432.454 (428.801)	209.936 (363.233)

### 3.2 Body image disturbance

**Figure 2:**
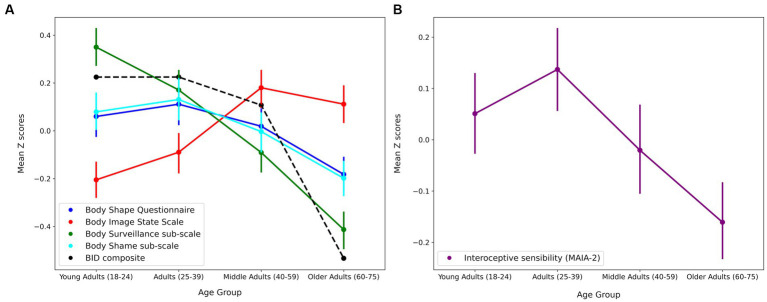
Mean *z*-standardized scores of the composite body image disturbance (BID) measure, including the aggregated scores from the body shape questionnaire, body image state scale, body surveillance, and shame sub-scales of the OBC **(A)** Mean *z*-standardised interoceptive sensibility scores from the MAIA-2 Questionnaire **(B)** Error bars indicate 95% confidence intervals. Data plotted across different age groups.

A between-groups ANOVA was conducted to compare the BID composite for each age group (see Table 1 for means and standard deviations). The ANOVA revealed a significant difference in BID composite scores between Age groups [*F* (3, 1,212) = 6.545, *p* < 0.001, η^2^*_p_* = 0.016]. Post-hoc comparisons indicate that there was significantly higher BID in the Young Adults group (*t* = 3.854, *SE* = 0.219, *p* < 0.001, *d* = 0.311), Adult group (*t* = 3.677, *SE* = 0.222, *p* = 0.001, *d* = 0.300) and Middle Adult group (*t* = 3.156, SE = 0.222, *p* = 0.01, *d* = 0.258) compared to the Older Adult group. There were no significant differences in BID between the Young Adult, Adult and Middle Adult groups. See Figure 2A to see the comparison of BID and body image scales across age groups.

### 3.3 Age-related differences across body image and interoceptive sensibility measures

Between-group ANOVAs were conducted to investigate age-related variances in body image and IS. Full reported means and standard deviations are presented in Table 1. *Z*-scores for body image measures are plotted in Figure 2A and *Z*-scores for IS are plotted in Figure 2B.

#### 3.3.1 Body shape questionnaire

The ANOVA revealed significant age group differences in the BSQ [*F* (3, 1,212) = 12.782, *p* < 0.001, η^2^_p_ = 0.016]. Post-hoc comparisons indicate significantly higher BSQ scores in the Young Adults group compared to the Older Adults group (*t* = 3.569, *SE* = 2.620. *p* = 0.002, *d* = 0.288). Significantly higher BSQ scores were also reported in the Adult group (*t* = 3.950, *SE* = 2.648, *p* < 0.001, *d* = 0.322) and the Middle Adult group (*t* = 2.816, *SE* = 2.655, *p* = 0.03, *d* = 0.230) compared to the Older Adult group. There were no significant differences in BSQ scores between the Young Adult, Adult and Middle Adult age groups.

#### 3.3.2 Body image state scale

The ANOVA revealed significant age-group differences in the BISS [*F* (3, 1,212) = 16.271, *p* < 0.001, η^2^_p_ = 0.020]. Post-hoc comparisons indicate significantly lower BISS scores in the Young Adults group compared to the Middle Adults group (*t* = −4.399, *SE* = 0.784, *p* < 0.001, *d* = −0.356) and the Older Adult group (*t* = −3.299, *SE* = 0.782, *p* = 0.006, *d* = −0.274). Significantly lower BISS scores were also reported in the Adult group compared to the Middle Adult group (*t* = −3.353, *SE* = 0.792, *p* = 0.005, *d* = −0.274).

#### 3.3.3 Body surveillance

The ANOVA revealed significant age group differences in the Body Surveillance Subscale [*F* (3, 1,202) = 70.791, *p* < 0.001, η^2^_p_ = 0.018]. Post-hoc comparisons highlighted significantly higher body surveillance scores in the Young Adult group compared to the Middle Adult group (*t* = 5.366, *SE* = 0.711, *p* < 0.001, *d* = 0.434) and the Older adult group (*t* = 9.642, *SE* = 0.709, *p* < 0.001, *d* = 0.778). Significantly higher surveillance scores were also reported in the Adult group compared to the Middle (*t* = 3.329, *SE* = 0.719, *p* = 0.005, *d* = 0.272) and Older Adult group (*t* = 7.555, *SE* = 0.717, *p* < 0.001, *d* = 0.616). Surveillance scores were also significantly higher in the Middle Adult group compared to the Older Adult group (*t* = 4.207, *SE* = 0.719, *p* < 0.001, *d* = 0.344).

#### 3.3.4 Body shame

The ANOVA revealed significant age group differences in the Body Shame subscale [*F* (3, 1,210) = 7.433, *p* < 0.001, η^2^_p_ = 0.017]. Post-hoc comparisons highlighted significantly higher body shame scores in the Young Adult (*t* = 3.841, *SE* = 0.714, *p* < 0.001, *d* = 0.310), Adult (*t* = 4.311, SE = 0.721, *p* < 0.001, *d* = 0.351) and Middle Adult (*t* = 2.665, SE = 0.723, *p* = 0.047, *d* = 0.310) groups compared to the Older Adult group. There were no significant differences in body shame scores between the Young Adult, Adult and Middle Adult groups.

#### 3.3.5 Interoceptive sensibility

The ANOVA revealed a significant difference in scores on the MAIA-2 between the age groups [*F* (3, 1,210) = 4.839, *p* = 0.002, η^2^_p_ = 0.012]. Post-hoc comparisons indicated that this difference was in higher IS scores in the Adult group compared to the Older Adult group (*t* = 3.704, *SE* = 1.328, *p* = 0.001, *d* = 0.302) (see Figure 2B). No other statistically significant group differences in IS were found.^4^

### 3.4 The effect of interoceptive sensibility and age on body image disturbance

**Figure 3:**
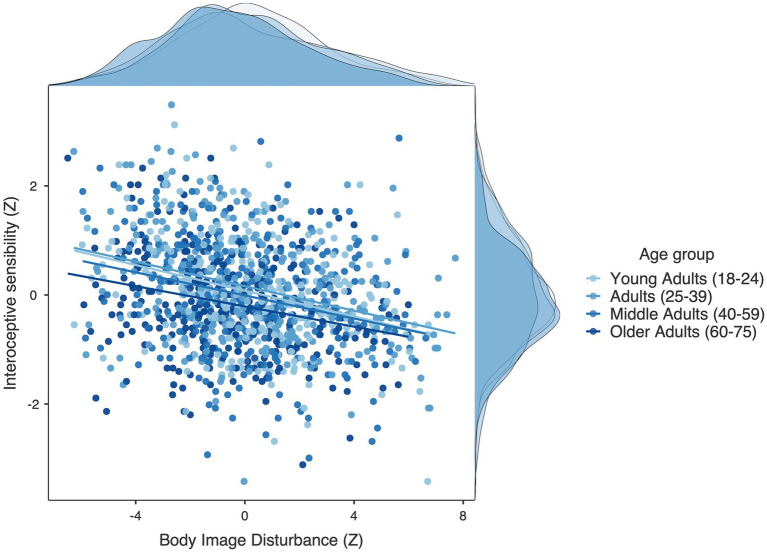
Scatter plot showing *z* standardised Interoceptive sensibility scores on the *Y* axis and body image disturbance on the *X* axis with regression line plotted for each age group. Marginal densities for interoceptive sensibility and body image disturbance are displayed on the sides of the graph.

To examine the contribution of age and interoception on body image disturbance a multiple hierarchical regression was conducted with the composite measure of BID as the outcome and age group and IS as predictors. The different age groups (Adults, Middle Adults and Older adults) were entered into the null model, with Young Adults as the reference group. The first step was found to be significant [*R*^2^ = 0.0167, *F* (3, 1,212) = 6.545, *p* < 0.001]. Older Age was a significant negative predictor of BID (*SE* = 0.184, *p* < 0.001). IS was then entered into the second step of the model, this was found to be a significant contribution to the model [R^2^ = 0.066, *F* (4, 1,211) = 34.067, *p* < 0.001]. IS was a significant negative predictor of overall BID (*t* = −10.714, *p* < 0.001) (See Figure 3) and each individual subscale of BID (see Supplementary files S1, S2 for full reported regression results and correlation matrix of IS and all body image questionnaires).

### 3.5 Own body transformation task

#### 3.5.1 Performance

Overall performance was above chance with overall mean accuracy (% correct) (*M* = 80.97, *SD* = 16.67), and overall Mean RT (*M* = 1783.70 ms, *SD* = 765.62). Please see Table 2 for full descriptive statistics for this task. Of note, accuracy for side-facing experimental trials was low and barely above chance level (*M* = 51.33, *SD* = 8.83). Due to this high error rate side-facing trials were subsequently excluded from further analysis, deviating from the initial pre-registration plan.

#### 3.5.2 Experimental vs. control

To examine the efficacy of the experimental versus control conditions we ran a 2 * 2 ANOVA with Condition (*control, experimental*) and Positio*n* (*front-facing, back-facing*) on 0- degree trials as within subjects’ factors and Accuracy (% correct) as the dependent variable. As expected, a main effect of Condition [*F*(1,4,828) = 294.83, *p* < 0.001, η^2^_p_ = 0.058] and Position [*F* (1, 4,828) = 6.133, *p* = 0.013, η^2^_p_ = 0.001] emerged. There was no significant Position*Condition interaction on accuracy [*F*(1,4,828) = 0.30, *p* = 0.584, η^2^*_p_* < 0.001]. Post-hoc tests indicate significantly higher accuracy for control trials (*M* = 94.89, *SD* = 15.84) compared to experimental trials (*M* = 93.89, *SD* = 12.86) (*t* = 2.477, *SE* = 0.403, *p* = 0.013, *d* = 0.07) in all positions except for back-facing trials (for full *post hocs* see Supplementary file S3).

#### 3.5.3 OBT performance

To investigate performance on the OBT task we calculated Mean RT on correct trials for Orientation, Weight, and Posture of avatar per Age group and ran a 2x2x2x4 omnibus mixed-model ANOVA with Posture (front-facing, back-facing), Orientation (0 degrees, 90 degrees), and Weight (underweight, overweight) as and Age group (Young Adults, Adults, Middle-Adults, Older adults) as the between-subjects factor. The analysis revealed a significant main effect of Position [*F* (1,1,262) = 8.6831, *p* = 0.003, η^2^_p_ = 0.007] on RT. Post-hoc comparisons show that back-facing trials (*M* = 1,499 ms, *SD* = 539) had faster reaction times compared to front-facing trials (*M* = 1864 ms, *SD* = 619) (*t* = −2.95, SE = 0.109, *p* = 0.003, *d* = −0.021). However, there were no significant interactions between Position and Age group indicating that all age groups demonstrated comparable RTs in trials across position of avatar. There was also no significant main effect of Orientation [*F* (1,1,262) = 0.0106, *p* = 0.918, η^2^_p_ < 0.000]. While there was a significant interaction effect between Orientation and Age group [*F* (3,1,262) = 2.8009, *p* = 0.039, η^2^_p_ = 0.007], *post-hoc t*-tests did not survive Bonferroni corrections. There were no significant main effects of Weight [*F* (1,1,262) = 0.3998, *p* = 0.527 η^2^_p_ = 0.000]. There were no significant two or three-way interactions and no significant between-subjects effects of age (see Supplementary file S4 for full reported ANOVA results, post-hocs and interactions).

#### 3.5.4 Egocentric transformation cost

**Figure 4:**
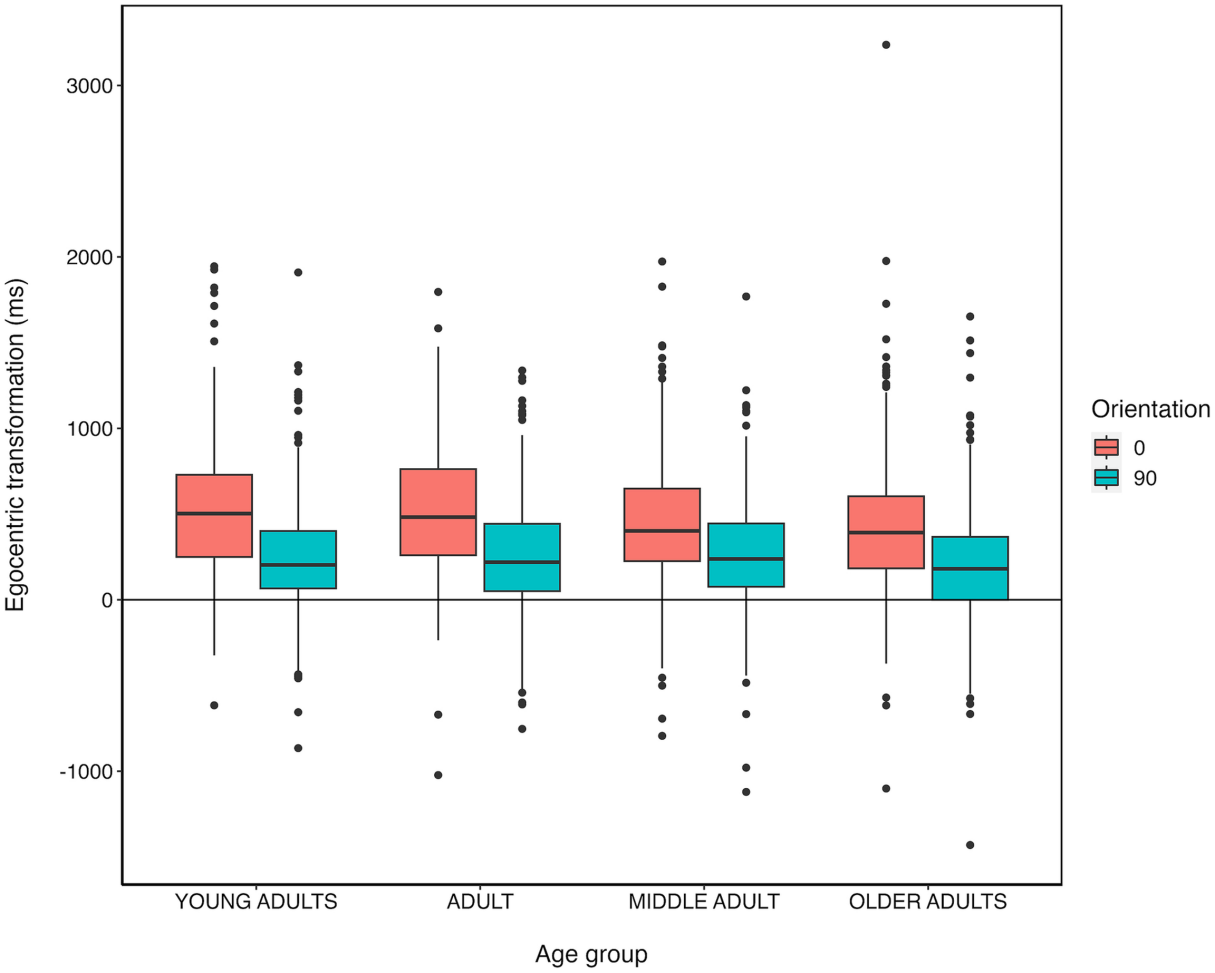
Boxplots indicating the mean reaction time difference (ms) for egocentric transformations (front-facing -back-facing) (*Y* axis) by age group (*X* axis), separated by orientation (0 and 90 degrees).

On the OBT task participants typically demonstrate an egocentric transformation effect, exemplified by prolonged reaction times when mentally aligning front-facing avatars with their own egocentric view, as opposed to back-facing avatars (Zacks et al., 1999; Gardner et al., 2012). From the outcome of the above ANOVA (See 3.5.3), participants exhibited an egocentric transformation effect (faster performance for back-facing trials than front-facing trials). To capture this transformation cost, an egocentric transformation index was calculated by subtracting Mean RT on correct trials for the position of the avatar [front-facing - back-facing]. As avatar weight had no effect on RTs as reported above, weight was dropped as a variable from the analysis. Mean egocentric transformation scores across age groups and orientations are reported in Table 2 and can be seen in Figure 4.

A two-way ANOVA was conducted to investigate differences in egocentric transformation cost across the four age groups between different Orientations. The analysis revealed a significant difference in egocentric transformation cost [*F* (3, 2,373) = 3.541, *p* = 0.014, η^2^_p_ = 0.004]. Post-hoc tests indicate significantly lower transformation cost scores in the Older Adult group (*M* = 321.01 ms, *SD* = 412.30) compared to the Young Adult group (*M* = 383.49 ms, *SD* = 382.77) (*t* = 2.946, *SE* = 21.365, *p* = 0.019, *d* = 0.170). The analysis also revealed significant differences in transformation cost across orientation [*F* (1, 2,373) = 242.54, *p* < 0.001, η^2^_p_ = 0.093]. *Post hoc* tests indicate that participants had larger egocentric transformation cost at 0 degrees (*M* = 477.30 ms, SD = 394.47) compared to 90 degrees (*M* = 240.18 ms, *SD* = 347.22) (*t* = 15.574, *SE* = 15.202, *p* < 0.001, *d* = 0.639). There was no significant interaction between age group and Orientation on the egocentric transformation cost [*F* (3, 2,373) =1.753, *p* = 0.158, η^2^_p_ = 0.002].

#### 3.5.5 Does BID and IS predict egocentric mental transformation performance?

A multiple hierarchical regression model was conducted to investigate the effect of BID and IS on the rotational cost of making an egocentric transformation [front-facing RTs - back-facing RTs]. Orientation of avatar (0 degrees, 90 degrees) and Age group (Young Adult, Adults, Middle Adults, Older Adults) were entered as factors (due to the significant effects of orientation and age on the egocentric transformation cost) in the null model with the young adults and 0 degrees as the reference group. The first step was found to be significant [*R*^2^ = 0.097, *F* (2,2,378) = 126.92, *p* < 0.001]. Age and Orientation accounted for 9% of the total variance in the egocentric transformation cost. The oldest age group was a significant negative predictor of the model (*SE* = 21.362, *p* = 0.003) and 90-degree orientation was also a significant negative predictor of the model (*SE* = 15.188, *p* < 0.001). Then BID and IS were entered into the second step of the model. The model was significant [*R*^2^ = 0.099, *F* (4, 2,376) = 65.276, *p* < 0.001], with a significant model change over the null model (*p* < 0.05). Overall, the model showed that Orientation (*SE* = 177.238, *p* < 0.001) and BID (*SE* = 3.29, *p* = 0.016) were significant positive predictors of the transformation cost. IS did not emerge as a significant predictor of the model (unstandardized coefficients for the hierarchical linear regression analysis for variables predicting the egocentric transformation cost can be found in Supplementary file S5). An exploratory correlational analysis was conducted to investigate the relationship between individual BID components and back-facing and front-facing RTs in 0- or 90-degree avatar orientations to investigate the relationship between the variables further (see Supplementary file S6 for correlation matrix). Pearson’s correlation coefficient indicated that in the 90-degree condition, body shame and back-facing rotations were positively correlated *r* (1190) = 0.059, *p* = 0.04. And body shape concern and front-facing rotations were strongly positively correlated *r* (1190) = 0.095, *p* < 0.001. Additionally, BISS scores and front-facing 90-degree rotations were strongly related *r* (1190) = 0.083, *p* = 0.004.

## 4 Discussion

The aim of the current study was firstly, to examine body image disturbance, interoceptive sensibility and its pattern of expression across distinct epochs of female adulthood. Secondly, to investigate if body image disturbance and/or interoceptive sensibility predicts performance on an embodied motor imagery task that indexes the body schema.

### 4.1 Age-related differences in body image disturbance

With regards to the age-related variation of attitudinal negative body image: the BID composite scores remained comparable in the Young Adult, Adult and Middle Adult age groups, after which it significantly declined in the Older Adult group. Other body image trajectory and cohort studies support this finding, indicating that negative body image seems to be stable across female adulthood; specifically, from young (Ages: 18+) to the end of middle adulthood (Age: 60) after which it steadily declines into older adulthood (Tiggemann and McCourt, 2013; Fallon et al., 2014; Quittkat et al., 2019; Lacroix et al., 2023). However, to date, no study has looked at age-related differences across multiple different components of BID.

Upon examining the various components of BID, no significant differences were observed in body shame and body shape concern across the Young Adult, Adult, and Middle Adult groups. However, a notable decline was observed in the Older Adult group. The findings suggest that body shape and shame concerns in middle adulthood are comparable to those in young adulthood. While previous studies have suggested that individuals become less susceptible to body image concerns as they age (e.g., Feingold and Mazzella, 1998), it is important to recognize that women undergo various biopsychosocial changes throughout adulthood, such as pregnancy, childbirth, and menopause, which can affect the physical appearance of their bodies (Samuels et al., 2019). These changes contribute to the persistence of negative body image into middle adulthood, challenging the notion that young adulthood alone is a vulnerable period for body concerns and eating disorders. Indeed, research suggests that the perimenopausal period presents a risk factor for the initial onset or recurrence of an ED (Kilpela et al., 2015). Lewis and Cachelin (2001) found that middle-aged women (ages 50–65) exhibited higher levels of bulimic tendencies, drive for thinness, and reduced interoceptive awareness compared to older women (ages 66+) (Lewis and Cachelin, 2001). When considering the findings of the current study in conjunction with previous research, it is evident that women in middle adulthood may face similar risks and consequences associated with BIDs as younger women.

Women in the older age group demonstrated significantly lower body shame, body shape concern, and self-surveillance than women in the three younger age groups. This aligns with previous research findings suggesting that there is a notable decrease in negative body image in older adulthood (Öberg and Tornstam, 2001; Tiggemann and Lynch, 2001; Tiggemann and McCourt, 2013). One potential explanation for this is the shift from an appearance-focused evaluation in early to mid-adulthood to an emphasis on the functionality of one’s body in older age (Reboussin et al., 2000; Augustus-Horvath and Tylka, 2011). For instance, a study by Baker and Gringart (2009) found that physical health and fitness exerted a greater influence on body satisfaction and body-related self-esteem in older adults compared to physical appearance (Baker and Gringart, 2009). This suggests that various aspects of positive body image (e.g., body functionality) become more influential in the evaluation of the body during the later stages of adulthood.

Interestingly, when considering state body dissatisfaction, as measured by the BISS, middle and older adults exhibited higher levels of state body dissatisfaction compared to the younger age groups. This was a surprising and unexpected finding. However, state body image has not yet been investigated in older women within the current literature. One possible explanation for this novel finding - which shows the opposite direction across age groups compared to the three other measure of negative body image that we employed - is the influence of intra-individual variability that is known to have an impact on immediate self-evaluative states, such as feelings of self-worth and self-esteem (Paradise and Kernis, 2002). Additionally, physical aspects like BMI, are known to influence body image state (Melnyk et al., 2004; Rudiger et al., 2007). In middle adulthood, there is commonly an upward trend in BMI and body fat distribution, with significant increases during the menopausal period (Runfola et al., 2013; Kodoth et al., 2022). This can occur due to decreases in estrogen, which can impact body composition and slow down metabolism (Kodoth et al., 2022). Research conducted on middle-aged and older women consistently demonstrates a relationship between menopause, self-esteem, body dissatisfaction, and higher BMI (Algars et al., 2009; Ayers et al., 2010). Furthermore, research indicates that middle and older adult women have generally lower levels of self-esteem than younger adult women (Robins and Trzesniewski, 2005) and this has also been linked to body dissatisfaction in these age groups (Paa and Larson, 1998). As such, the comparable increase in state-level body dissatisfaction in the mid and older age group of women may be influenced by various factors, including hormonal changes during midlife that contribute to the deviation from the prevailing thin-young ideal standard of beauty(Kilpela al., 2015).

Age-related differences were also observed in body surveillance, whereby the young adult group showed significantly higher levels compared to all other age groups. Furthermore, there was a consistent linear trend of declining body surveillance with increasing age. This finding is in line with research that has reported that self-surveillance peaks in young adulthood (Ages:20–30) (Tiggemann and Lynch, 2001) and subsequently declines with increasing age (Tiggemann and Lynch, 2001; Greenleaf, 2005). Younger women are said to be more susceptible to societal influences on body image compared to older women (Kilpela et al., 2015). This vulnerability is associated with various youth behaviours, including increased social networking (Brajdić Vuković et al., 2018), formation of new peer networks (Dohnt and Tiggemann, 2006), and engagement in body talk (Wang et al., 2019). These findings support the claim that younger women exhibit specific vulnerabilities regarding body image, placing them at a higher risk of developing EDs compared to older age groups (Kilpela et al., 2015).

### 4.2 Interoceptive sensibility and body image disturbance across age

We observed a linear decline in IS from adulthood to middle adulthood and older age. Notably, a significant decrease in IS was observed specifically from adulthood to older adulthood. Older adulthood is associated with decreased interoceptive processing, attributed to age-related changes in nerve myelination and conduction (Verdú et al., 2000). This leads to reduced sensory discrimination and diminished processing of visceral signals in the body (Murphy et al., 2018; Palve and Palve, 2018). Current research supports the idea of reduced egocentric processing (e.g., self-body recognition) in older age, and this has also been linked to negative body image (Bellard et al., 2022). In the current study, we found that IS contributed a unique variance to BID independent of any age-related declines in IS. This novel finding provides evidence for the distinctive contribution of IS to BID, beyond recognized protective body image factors such as age (Samuels et al., 2019). Indeed, upon examining the association between individual body image scores and IS scores, while controlling for age, a consistent negative relationship was observed across all scales. This was similarly demonstrated by Todd et al. (2019a) who reported a negative correlation between interoceptive sensibility and aspects of both negative and positive body image in adolescents (Todd et al., 2019b). Overall, the findings of this study add to the growing evidence that interoception is a crucial contributing factor to BID: with the present study demonstrating this relationship throughout several stages of female adulthood.

### 4.3 The influence of BID, IS and age on the body schema

In the current study, the body schema was indexed by an embodied transformation task that requires motor imagery to complete (Blanke et al., 2005; Gardner et al., 2012). We demonstrate, for the first time, that BID in a healthy female population predicts a significant increase in the time taken to make an egocentric mental transformation. Difficulties in egocentric mental transformation have previously been reported in individuals with clinical levels of BIDs (e.g., individuals with EDs) (Urgesi et al., 2011; Cooper and Mohr, 2012; Serino et al., 2015; Cipolletta et al., 2017). In those studies, performance is compared to a non-ED control group, with poorer performance by the ED group suggesting that high levels of BID are associated with disruptions in the body schema. Here we show that high levels of BID in women without EDs are related to how they imagine their body via motor imagery. Previous findings in non-clinical populations have indicated that factors such as dietary restraint (Wignall et al., 2017) and body dissatisfaction (Irvine et al., 2019) contribute to an altered experience of the body schema. However, we found that a combination of different, equally weighted components of BID, contributes to alterations in how egocentric information is processed in relation to the body schema. The Allocentric Lock Theory supports the idea that BID impairs the utilization of real-time egocentric information (Riva, 2014). This has typically been observed in individuals with EDs, who show difficulties in constructing and updating an allocentric representation based on visual input, indicating a deficiency in using the egocentric reference frame (Serino et al., 2015; Lander et al., 2020). In this study, we successfully replicate this in individuals without EDs across adulthood: indicating that BID outside of clinical disorders can also influence the body schema.

However, contrary to the assertions of the Allocentric Lock Theory we found that egocentric sensory information, specifically interoception, did not significantly predict the time taken to make an egocentric transformation. In this study, our interoception (Interoceptive sensibility) measure was the MAIA-2, which measures explicit self-reported evaluations of one’s tendency to notice and attend to internal physiological states (Mehling et al., 2018). Interoception involves the transmission of signals between the central nervous system and the visceral organs (Vaitl, 1996) and denotes the ability to sense, perceive, and regulate internal visceral states (Sherrington, 1906; Chen et al., 2021). Hence, it is important to include psychophysiological assessments of visceral-afferent signal transmission (e.g., heartbeat discrimination, gastric signalling sensitivity) when investigating interoception (Forkmann et al., 2016). Indeed, a study by Baumann et al. (2022) found that perception of gastric signalling, a measure of IS, was related to body schema judgments in a body-scaled action task (Baumann et al., 2022). Given the observed association in the present study between explicit IS and explicit negative body image measures, future research could explore the relationship between implicit interoceptive processing and implicit body schema. The relationship between the body schema, an often-considered unconscious body representation, and interoception, a multidimensional construct, could be revisited using comprehensive measures of interoceptive propensity (Murphy, 2023).

Additionally, the analysis revealed that the age of the participants as well as the orientation of the stimuli were significant contributors to the egocentric transformation cost. The oldest age group demonstrated a significant negative contribution to the transformation cost, indicating that older women took a similar time to perform front-facing and back-facing mental rotations. This finding is consistent with research that indicates that ageing can lead to deficits in strategy switching in spatial tasks (Harris et al., 2012; Harris and Wolbers, 2014). Previous research that has employed motor imagery tasks such as the hand laterality task has reported that older adults show less of a distinction in performance between back-facing and palm-facing positions of the hand compared to younger adults (Nagashima et al., 2021). However, in the present study, all age groups performed similarly in terms of response accuracy. This is in line with research by Inagaki et al. (2002) who noted that the ability to rotate images is relatively preserved with age, leading to correct responses in spatial tasks, although the ability to shift perspective declines (Inagaki et al., 2002).

Furthermore, stimulus orientation also significantly influenced the cost of egocentric transformation. Specifically, the transformation costs were smaller for orientations at 90 degrees compared to 0 degrees. This finding can be attributed to biomechanical constraints affecting mental rotations of anatomically familiar versus unfamiliar orientations (Ionta et al., 2012). Anatomically familiar orientations (0 degrees) are typically processed faster than unfamiliar orientations (90 degrees), resulting in a smaller difference in the transformation cost between back-facing and front-facing conditions in the 90-degree orientation (Qu et al., 2018). Upon inspecting the association between the components of BID and the different orientation conditions of the OBT, we found that body shape concern, body image state and body shame were significantly positively correlated with the reaction times of performing egocentric mental rotations in 90 degrees as opposed to 0 degrees. This finding aligns with those reported by Wignall et al. (2017), who showed that non-clinical participants exhibit less representations of body width when imagining their bodies horizontally but not vertically (Wignall et al., 2017). Taken together, this indicates that pre-clinical BID is associated with the ability to make egocentric transformations when the body is imagined in orientations incongruent to familiar embodied body processing.

## 5 Limitations and future directions

It is important to note that although BID contributes to the time taken to make an embodied transformation, much of the variance in transformation time remains unaccounted for even after factoring in age and interoception. Therefore, self-report BID and IS are not sufficient alone to explain the performance in the OBT task in individuals without EDs. Our sample comprises participants from many different countries, suggesting ethnicity as a relevant covariate. However, body image has now become a global health concern, accelerated by the rapid technological and economic globalization (Thornborrow et al., 2022), which has resulted in a convergence of appearance concerns among women across the world (Rodgers et al., 2023). While our study did not examine the impact of ethnicity on body image, we strongly advocate for future research to explore the role of race and to incorporate considerations of minority stress and body capital theories (Rodgers et al., 2023) when investigating the relationship between BID, IS and the body schema. Lastly, studies using a cross-sectional design are prone to cohort effects (Glenn, 2005) and are more reflective of age-related differences as opposed to age-related developmental changes. Hence, future research should incorporate a longitudinal approach to investigate the developmental trajectory of BID from early adolescence to late adulthood. Understanding how BID emerges in adolescence will allow us to explore causal relationships regarding its effects on interoception and body schema.

## 6 Conclusion

The present study demonstrated: (a) that components of body image disturbance take on different trajectories across female adulthood, and (b) a disturbance in body image is associated with how the body schema is manipulated in non-clinical populations. These findings are important given that there is little research on how different facets of body image and the body schema develop in relation to one another across the female lifespan. Overall, these findings suggest that disruptions in body image have the potential to impact the body schema even in non-clinical populations while considering important individual factors such as age.

## Data availability statement

The datasets presented in this study can be found in online repositories. The names of the repository/repositories and accession number(s) can be found at: https://osf.io/jn48r/?view_only=55e256276f574b73825432c7f22c2fb6.

## Ethics statement

The studies involving humans were approved by the Human Research Ethics Committee, University College Dublin. The studies were conducted in accordance with the local legislation and institutional requirements. The participants provided their written informed consent to participate in this study.

## Author contributions

AN: Conceptualization, Data curation, Formal analysis, Investigation, Methodology, Visualization, Writing – original draft. SC: Conceptualization, Funding acquisition, Project administration, Supervision, Writing – review & editing.

## Footnotes

^1^https://www.prolific.co/

^2^https://osf.io/jn48r/

^3^https://pavlovia.org/

^4^After pre-registering this study in 2021, new recommendations suggest that the MAIA-2 total score should be calculated by averaging the scores of its sub-scales (Datko et al., 2022; Hübner et al., 2022; Millman et al., 2023). Considering this, we calculated IS using this method and ran the analyses again and observed no changes in the findings (see Supplementary file S8 for full reported result)

## References

[ref1] Ainley V. Tsakiris M. (2013). Body conscious? Interoceptive awareness, measured by heartbeat perception, is negatively correlated with self-objectification. PLoS One 8:e55568. doi: 10.1371/journal.pone.0055568, PMID: 23405173 PMC3565964

[ref2] Algars M. Santtila P. Varjonen M. Witting K. Johansson A. Jern P.. (2009). The adult body: how age, gender, and body mass index are related to body image. J. Aging Health 21, 1112–1132. doi: 10.1177/0898264309348023 19897779

[ref4] Andrade C. (2021). Z scores, standard scores, and composite test scores explained. Indian J. Psychol. Med. 43, 555–557. doi: 10.1177/02537176211046525, PMID: 35210687 PMC8826187

[ref5] Augustus-Horvath C. L. Tylka T. L. (2011). The acceptance model of intuitive eating: a comparison of women in emerging adulthood, early adulthood, and middle adulthood. J. Couns. Psychol. 58, 110–125. doi: 10.1037/a0022129 21244144

[ref6] Ayers B. Forshaw M. Hunter M. S. (2010). The impact of attitudes towards the menopause on women's symptom experience: a systematic review. Maturitas 65, 28–36. doi: 10.1016/j.maturitas.2009.10.016 19954900

[ref7] Badoud D. Tsakiris M. (2017). From the body’s viscera to the body’s image: is there a link between interoception and body image concerns? Neurosci. Biobehav. Rev. 77, 237–246. doi: 10.1016/j.neubiorev.2017.03.017 28377099

[ref8] Baker L. Gringart E. (2009). Body image and self-esteem in older adulthood. Ageing Soc. 29, 977–995. doi: 10.1017/S0144686X09008721

[ref9] Baumann P. Beckmann N. Herpertz S. Trojan J. Diers M. (2022). Influencing the body schema through the feeling of satiety. Sci. Rep. 12:2350. doi: 10.1038/s41598-022-06331-3, PMID: 35149735 PMC8837638

[ref10] Becker A. E. (2004). Television, disordered eating, and young women in Fiji: negotiating body image and identity during rapid social change. Cult. Med. Psychiatry 28, 533–559. doi: 10.1007/s11013-004-1067-5 15847053

[ref12] Beckmann N. Baumann P. Herpertz S. Trojan J. Diers M. (2021). How the unconscious mind controls body movements: body schema distortion in anorexia nervosa. Int. J. Eat. Disord. 54, 578–586. doi: 10.1002/eat.23451, PMID: 33345338

[ref13] Bellard A. Urgesi C. Cazzato V. (2022). Self-body recognition and attitudes towards body image in younger and older women. Arch. Womens Ment. Health 25, 107–119. doi: 10.1007/s00737-021-01164-x, PMID: 34331575 PMC8784361

[ref14] Blanke O. Mohr C. Michel C. M. Pascual-Leone A. Brugger P. Seeck M.. (2005). Linking out-of-body experience and self processing to mental own-body imagery at the Temporoparietal junction. J. Neurosci. 25, 550–557. doi: 10.1523/JNEUROSCI.2612-04.2005 15659590 PMC6725328

[ref15] Brajdić Vuković M. Lucić M. Stulhofer A. (2018). Internet use associated body-surveillance among female adolescents: assessing the role of peer networks. Sex. Cult. 22, 521–540. doi: 10.1007/s12119-017-9480-4

[ref16] Bujang M. A. Sa’at N. Sidik T. M. I. T. A. B. Joo L. C. (2018). Sample size guidelines for logistic regression from observational studies with large population: emphasis on the accuracy between statistics and parameters based on real life clinical data. Malays. J. Med. Sci. 25, 122–130. doi: 10.21315/mjms2018.25.4.12, PMID: 30914854 PMC6422534

[ref18] Carrard I. Rothen S. Rodgers R. F. (2021). Body image concerns and intuitive eating in older women. Appetite 164:105275. doi: 10.1016/j.appet.2021.105275, PMID: 33915210

[ref19] Cash T. F. Deagle E. A. (1997). The nature and extent of body-image disturbances in anorexia nervosa and bulimia nervosa: a meta-analysis. Int. J. Eat. Disord. 22, 107–126. doi: 10.1002/(sici)109108x(199709)22:2<107::aid-eat1>3.0.co;2-j, PMID: 9261648

[ref20] Cash T. F. Fleming E. C. Alindogan J. Steadman L. Whitehead A. (2002). Beyond body image as a trait: the development and validation of the body image states scale. Eat. Disord. 10, 103–113. doi: 10.1080/10640260290081678, PMID: 16864251

[ref21] Cash T. F. Morrow J. A. Hrabosky J. I. Perry A. A. (2004). How has body image changed? A cross-sectional investigation of college women and men from 1983 to 2001. J. Consult. Clin. Psychol. 72, 1081–1089. doi: 10.1037/0022-006X.72.6.1081, PMID: 15612854

[ref22] Chen W. G. Schloesser D. Arensdorf A. M. Simmons J. M. Cui C. Valentino R.. (2021). The emerging science of interoception: sensing, integrating, interpreting, and regulating signals within the self. Trends Neurosci. 44, 3–16. doi: 10.1016/j.tins.2020.10.007 33378655 PMC7780231

[ref23] Cipolletta S. Malighetti C. Serino S. Riva G. Winter D. (2017). Intrapersonal, interpersonal, and physical space in anorexia nervosa: a virtual reality and repertory grid investigation. Psychiatry Res. 252, 87–93. doi: 10.1016/j.psychres.2017.02.060 28259036

[ref24] Colautti L. A. Fuller-Tyszkiewicz M. Skouteris H. McCabe M. Blackburn S. Wyett E. (2011). Accounting for fluctuations in body dissatisfaction. Body Image 8, 315–321. doi: 10.1016/j.bodyim.2011.07.001, PMID: 21840778

[ref25] Cooper K. Mohr C. (2012). Former eating disorder impairs 3rd person but not 1st person perspective taking: does dance training help? Comp.Psychol. 1:02.06.20.CP.1.7. doi: 10.2466/02.06.20.CP.1.7

[ref26] Cooper P. J. Taylor M. J. Cooper Z. Fairbum C. G. (1987). The development and validation of the body shape questionnaire. Int. J. Eat. Disord. 6, 485–494. doi: 10.1002/1098-108X(198707)6:4<485::AID-EAT2260060405>3.0.CO;2-O

[ref27] Coslett H. B. Medina J. Kliot D. Burkey A. R. (2010). Mental motor imagery indexes pain: the hand laterality task. Eur. J. Pain 14, 1007–1013. doi: 10.1016/j.ejpain.2010.04.001, PMID: 20638306 PMC2958243

[ref28] Costello M. C. Bloesch E. K. (2017). Are older adults less embodied? A review of age effects through the Lens of embodied cognition. Front. Psychol. 8:267. doi: 10.3389/fpsyg.2017.00267, PMID: 28289397 PMC5326803

[ref29] Craig A. D. (2002). How do you feel? Interoception: the sense of the physiological condition of the body. Nat. Rev. Neurosci. 3, 655–666. doi: 10.1038/nrn894 12154366

[ref30] Datko M. Lutz J. Gawande R. Comeau A. To M. N. Desel T.. (2022). Increased insula response to interoceptive attention following mindfulness training is associated with increased body trusting among patients with depression. Psychiatry Res. Neuroimaging 327:111559. doi: 10.1016/j.pscychresns.2022.111559 36308976 PMC12981234

[ref31] de Vignemont F. (2010). Body schema and body image—pros and cons. Neuropsychologia 48, 669–680. doi: 10.1016/j.neuropsychologia.2009.09.022 19786038

[ref32] Dohnt H. K. Tiggemann M. (2006). Body image concerns in young girls: the role of peers and media prior to adolescence. J. Youth Adolesc. 35, 135–145. doi: 10.1007/s10964-005-9020-7

[ref33] Emanuelsen L. Drew R. Köteles F. (2015). Interoceptive sensitivity, body image dissatisfaction, and body awareness in healthy individuals. Scand. J. Psychol. 56, 167–174. doi: 10.1111/sjop.12183, PMID: 25444023

[ref34] Eshkevari E. Rieger E. Musiat P. Treasure J. (2014). An investigation of interoceptive sensitivity in eating disorders using a heartbeat detection task and a self-report measure. Eur. Eat. Disord. Rev. 22, 383–388. doi: 10.1002/erv.2305 24985151

[ref1001] Erbil N. (2018). Attitudes towards menopause and depression, body image of women during menopause. Alexandria Journal of Medicine, 54, 241–246. doi: 10.1016/j.ajme.2017.05.012

[ref35] Fallon E. A. Harris B. S. Johnson P. (2014). Prevalence of body dissatisfaction among a United States adult sample. Eat. Behav. 15, 151–158. doi: 10.1016/j.eatbeh.2013.11.007 24411768

[ref36] Feingold A. Mazzella R. (1998). Gender differences in body image are increasing. Psychol. Sci. 9, 190–195. doi: 10.1111/1467-9280.00036

[ref37] Forbes G. B. Jobe R. L. Revak J. A. (2006). Relationships between dissatisfaction with specific body characteristics and the sociocultural attitudes toward appearance Questionnaire-3 and objectified body consciousness scale. Body Image 3, 295–300. doi: 10.1016/j.bodyim.2006.07.003, PMID: 18089232

[ref38] Forkmann T. Scherer A. Meessen J. Michal M. Schächinger H. Vögele C.. (2016). Making sense of what you sense: disentangling interoceptive awareness, sensibility and accuracy. Int. J. Psychophysiol. 109, 71–80. doi: 10.1016/j.ijpsycho.2016.09.019, PMID: 27702644

[ref39] Franko D. L. Jenkins A. Roehrig J. P. Luce K. H. Crowther J. H. Rodgers R. F. (2012). Psychometric properties of measures of eating disorder risk in Latina college women. Int. J. Eat. Disord. 45, 592–596. doi: 10.1002/eat.20979, PMID: 22271562

[ref40] Gagne D. A. Von Holle A. Brownley K. A. Runfola C. D. Hofmeier S. Branch K. E.. (2012). Eating disorder symptoms and weight and shape concerns in a large web-based convenience sample of women ages 50 and above: results of the gender and body image (GABI) study. Int. J. Eat. Disord. 45, 832–844. doi: 10.1002/eat.22030, PMID: 22729743 PMC3459309

[ref41] Gallagher S. (2005). How the body shapes the mind. Oxford, UK: Oxford University Press. doi: 10.1093/0199271941.001.0001

[ref42] Gardner M. R. Sorhus I. Edmonds C. J. Potts R. (2012). Sex differences in components of imagined perspective transformation. Acta Psychol. 140, 1–6. doi: 10.1016/j.actpsy.2012.02.002, PMID: 22426425

[ref43] Garfinkel S. N. Seth A. K. Barrett A. B. Suzuki K. Critchley H. D. (2015). Knowing your own heart: distinguishing interoceptive accuracy from interoceptive awareness. Biol. Psychol. 104, 65–74. doi: 10.1016/j.biopsycho.2014.11.004 25451381

[ref45] Glashouwer K. A. Meulman C. de Jong P. J. (2019). Negative body image is not related to spontaneous body-scaled motoric behavior in undergraduate women. Front. Psychol. 10:580. doi: 10.3389/fpsyg.2019.00580 30971972 PMC6443978

[ref46] Glenn N. D. (2005). “Age, Period, and Cohort Effects,” in Encyclopedia of Social Measurement ed. Kempf-Leonard K.. Amsterdam, The Netherlands: Elsevier. 27–32. doi: 10.1016/B0-12-369398-5/00113-4

[ref47] Gorrell S. Trainor C. Le Grange D. (2019). The impact of urbanization on risk for eating disorders. Curr. Opin. Psychiatry 32, 242–247. doi: 10.1097/YCO.0000000000000497, PMID: 30724753 PMC6438744

[ref48] Grabe S. Hyde J. S. Lindberg S. M. (2007). Body objectification and depression in adolescents: the role of gender, shame, and rumination. Psychol. Women Q. 31, 164–175. doi: 10.1111/j.1471-6402.2007.00350.x

[ref49] Greenleaf C. (2005). Self-objectification among physically active women. Sex Roles 52, 51–62. doi: 10.1007/s11199-005-1193-8

[ref50] Greenleaf C. McGreer R. (2006). Disordered eating attitudes and self-objectification among physically active and sedentary female college students. J. Psychol. 140, 187–198. doi: 10.3200/JRLP.140.3.187-198, PMID: 16916073

[ref51] Grippo K. P. Hill M. S. (2008). Self-objectification, habitual body monitoring, and body dissatisfaction in older European American women: exploring age and feminism as moderators. Body Image 5, 173–182. doi: 10.1016/j.bodyim.2007.11.003, PMID: 18458007

[ref53] Gronholm P. C. Flynn M. Edmonds C. J. Gardner M. R. (2012). Empathic and non-empathic routes to visuospatial perspective-taking. Conscious. Cogn. 21, 494–500. doi: 10.1016/j.concog.2011.12.004, PMID: 22221741

[ref54] Guardia D. Conversy L. Jardri R. Lafargue G. Thomas P. Dodin V.. (2012). Imagining One’s own and someone Else’s body actions: dissociation in anorexia nervosa. PLoS One 7:e43241. doi: 10.1371/journal.pone.0043241, PMID: 22937025 PMC3425562

[ref55] Guardia D. Lafargue G. Thomas P. Dodin V. Cottencin O. Luyat M. (2010). Anticipation of body-scaled action is modified in anorexia nervosa. Neuropsychologia 48, 3961–3966. doi: 10.1016/j.neuropsychologia.2010.09.004, PMID: 20833193

[ref56] Harris M. A. Wolbers T. (2014). How age-related strategy switching deficits affect wayfinding in complex environments. Neurobiol. Aging 35, 1095–1102. doi: 10.1016/j.neurobiolaging.2013.10.086 24239438

[ref57] Harris M. Wiener J. Wolbers T. (2012). Aging specifically impairs switching to an allocentric navigational strategy. Front. Aging Neurosci. 4:29. doi: 10.3389/fnagi.2012.00029, PMID: 23125833 PMC3485570

[ref58] Head H. Holmes G. (1911). Sensory disturbances from cerebral lesions. Brain 34, 102–254. doi: 10.1093/brain/34.2-3.102

[ref59] Heydrich L. Walker F. Blättler L. Herbelin B. Blanke O. Aspell J. E. (2021). Interoception and empathy impact perspective taking. Front. Psychol. 11:9429. doi: 10.3389/fpsyg.2020.599429, PMID: 33536971 PMC7848222

[ref60] Hockey A. Milojev P. Sibley C. G. Donovan C. L. Barlow F. K. (2021). Body image across the adult lifespan: a longitudinal investigation of developmental and cohort effects. Body Image 39, 114–124. doi: 10.1016/j.bodyim.2021.06.007 34271529

[ref61] Hübner A. M. Trempler I. Schubotz R. I. (2022). Interindividual differences in interoception modulate behavior and brain responses in emotional inference. NeuroImage 261:119524. doi: 10.1016/j.neuroimage.2022.119524, PMID: 35907498

[ref62] Inagaki H. Meguro K. Shimada M. Ishizaki J. Okuzumi H. Yamadori A. (2002). Discrepancy between mental rotation and perspective-taking abilities in normal aging assessed by Piaget’s three-mountain task. J. Clin. Exp. Neuropsychol. 24, 18–25. doi: 10.1076/jcen.24.1.18.969, PMID: 11935420

[ref63] Ionta S. Perruchoud D. Draganski B. Blanke O. (2012). Body context and posture affect mental imagery of hands. PLoS One 7:e34382. doi: 10.1371/journal.pone.0034382, PMID: 22479618 PMC3316677

[ref64] Irvine K. R. McCarty K. McKenzie K. J. Pollet T. V. Cornelissen K. K. Tovée M. J.. (2019). Distorted body image influences body schema in individuals with negative bodily attitudes. Neuropsychologia 122, 38–50. doi: 10.1016/j.neuropsychologia.2018.11.015, PMID: 30500663

[ref65] Jackson T. Chen H. (2015). Features of objectified body consciousness and sociocultural perspectives as risk factors for disordered eating among late-adolescent women and men. J. Couns. Psychol. 62, 741–752. doi: 10.1037/cou0000096, PMID: 26191981

[ref66] Team JASP. (2023). JASP (version 0.17.2)[computer software]. Available at: https://jasp-stats.org/

[ref67] Jeannerod M. (2001). Neural simulation of action: a unifying mechanism for motor cognition. NeuroImage 14, S103–S109. doi: 10.1006/nimg.2001.0832 11373140

[ref68] Jenkinson P. M. Taylor L. Laws K. R. (2018). Self-reported interoceptive deficits in eating disorders: a meta-analysis of studies using the eating disorder inventory. J. Psychosom. Res. 110, 38–45. doi: 10.1016/j.jpsychores.2018.04.005 29764604

[ref70] Kaltner S. Riecke B. E. Jansen P. (2014). Embodied mental rotation: a special link between egocentric transformation and the bodily self. Front. Psychol. 5:505. doi: 10.3389/fpsyg.2014.00505, PMID: 24917832 PMC4042493

[ref71] Karazsia B. T. Murnen S. K. Tylka T. L. (2017). Is body dissatisfaction changing across time? A cross-temporal meta-analysis. Psychol. Bull. 143, 293–320. doi: 10.1037/bul0000081 27893220

[ref72] Keizer A. Engel M. (2021). Body representation in anorexia nervosa. Open Sci. Framework. doi: 10.31219/osf.io/vep73

[ref73] Keizer A. Smeets M. A. M. Dijkerman H. C. Uzunbajakau S. van Elburg A. A. Postma A. (2013). Too fat to fit through the door: first evidence for disturbed body-scaled action in anorexia nervosa during locomotion. PLoS One 8:e64602. doi: 10.1371/journal.pone.0064602, PMID: 23734207 PMC3667140

[ref74] Khalsa S. S. Adolphs R. Cameron O. G. Critchley H. D. Davenport P. W. Feinstein J. S.. (2018). Interoception and mental health: a roadmap. Biol. Psychiatry. Cogn. Neurosci. Neuroimag. 3, 501–513. doi: 10.1016/j.bpsc.2017.12.004, PMID: 29884281 PMC6054486

[ref1002] Khalsa S. S. Rudrauf D. Tranel D. (2009). Interoceptive awareness declines with age. Psychophysiology, 46, 1130–1136. doi: 10.1111/j.1469-8986.2009.00859.x 19602175 PMC2865139

[ref76] Kilpela L. S. Becker C. B. Wesley N. Stewart T. (2015). Body image in adult women: moving beyond the younger years. Adv. Eat. Disord. 3, 144–164. doi: 10.1080/21662630.2015.1012728 26052476 PMC4452130

[ref77] Kodoth V. Scaccia S. Aggarwal B. (2022). The Sleep of the Ring: Comparison of the OURA Sleep Tracker Against Polysomnography. Behavioral Sleep Medicine. Mary Ann Liebert, Inc. 3, 573–581. doi: 10.1089/whr.2021.0119

[ref78] Lacroix E. Smith A. J. Husain I. A. Orth U. Von Ranson K. M. (2023). Normative body image development: a longitudinal meta-analysis of mean-level change. Body Image 45, 238–264. doi: 10.1016/j.bodyim.2023.03.003, PMID: 36965235

[ref79] Lander R. Heled E. Gur E. (2020). Executive functioning and spatial processing in anorexia nervosa: an experimental study and its significance for the allocentric lock theory. Eat. Weight Disord. 25, 1039–1047. doi: 10.1007/s40519-019-00728-2 31209765

[ref80] Leahey T. M. Crowther J. H. (2008). An ecological momentary assessment of comparison target as a moderator of the effects of appearance-focused social comparisons. Body Image 5, 307–311. doi: 10.1016/j.bodyim.2008.03.002 18585108

[ref81] Lewis D. M. Cachelin F. M. (2001). Body image, body dissatisfaction, and eating attitudes in midlife and elderly women. Eat. Disord. 9, 29–39. doi: 10.1080/106402601300187713 16864371

[ref82] Lindner D. Tantleff-Dunn S. Jentsch F. (2012). Social comparison and the ‘circle of objectification’. Sex Roles 67, 222–235. doi: 10.1007/s11199-012-0175-x

[ref83] Mangweth-Matzek B. Rupp C. I. Hausmann A. Assmayr K. Mariacher E. Kemmler G.. (2006). Never too old for eating disorders or body dissatisfaction: a community study of elderly women. Int. J. Eat. Disord. 39, 583–586. doi: 10.1002/eat.20327 17078123

[ref84] McKinley N. M. Hyde J. S. (1996). The objectified body consciousness scale: development and validation. Psychol. Women Q. 20, 181–215. doi: 10.1111/j.1471-6402.1996.tb00467.x

[ref85] McLaren L. Kuh D. (2004). Women’s body dissatisfaction, social class, and social mobility. Soc. Sci. Med. 58, 1575–1584. doi: 10.1016/S0277-9536(03)00209-0 14990360

[ref86] McLean S. A. Paxton S. J. Wertheim E. H. (2010). Factors associated with body dissatisfaction and disordered eating in women in midlife. Int. J. Eat. Disord. 43, 527–536. doi: 10.1002/eat.20737 19718668

[ref87] Mehling W. E. Acree M. Stewart A. Silas J. Jones A. (2018). The multidimensional assessment of interoceptive awareness, version 2 (MAIA-2). PLoS One 13:e0208034. doi: 10.1371/journal.pone.0208034, PMID: 30513087 PMC6279042

[ref88] Melnyk S. E. Cash T. F. Janda L. H. (2004). Body image ups and downs: prediction of intra-individual level and variability of women’s daily body image experiences. Body Image 1, 225–235. doi: 10.1016/j.bodyim.2004.03.003, PMID: 18089155

[ref89] Meregalli V. Tenconi E. Madan C. R. Somà E. Meneguzzo P. Ceccato E.. (2022). Beyond body image: what body schema and motor imagery can tell us about the way patients with anorexia nervosa experience their body. Psychiatry Clin. Neurosci. 77, 94–101. doi: 10.1111/pcn.13501, PMID: 36330847

[ref90] MillmanL. S. M.ShortE.StantonB.WinstonJ. S.NicholsonT. R.MehtaM. A.. (2023). Interoception in functional motor symptoms and functional seizures: preliminary evidence of intact accuracy alongside reduced insight and altered sensibility. Behav. Res. Ther. 168:104379. doi: 10.1016/j.brat.2023.104379, PMID: 37516011

[ref91] Mond J. Mitchison D. Latner J. Hay P. Owen C. Rodgers B. (2013). Quality of life impairment associated with body dissatisfaction in a general population sample of women. BMC Public Health 13:920. doi: 10.1186/1471-2458-13-920, PMID: 24088248 PMC3850528

[ref92] Murphy J. (2023). Interoception: where do we go from here? Q. J. Exp. Psychol. doi: 10.1177/17470218231172725, PMID: 37082986 PMC10798007

[ref93] Murphy J. Geary H. Millgate E. Catmur C. Bird G. (2018). Direct and indirect effects of age on interoceptive accuracy and awareness across the adult lifespan. Psychon. Bull. Rev. 25, 1193–1202. doi: 10.3758/s13423-017-1339-z, PMID: 28685271 PMC5990557

[ref94] Myers T. A. Crowther J. H. (2008). Is self-objectification related to interoceptive awareness? An examination of potential mediating pathways to disordered eating attitudes. Psychol. Women Q. 32, 172–180. doi: 10.1111/j.1471-6402.2008.00421.x

[ref95] Nagashima I. Takeda K. Harada Y. Mochizuki H. Shimoda N. (2021). Age-related differences in strategy in the hand mental rotation task. Front. Hum. Neurosci. 15:5584. doi: 10.3389/fnhum.2021.615584 PMC7987654 33776667

[ref96] Öberg P. Tornstam L. (2001). Youthfulness and fitness—identity ideals for all ages? J. Aging Identity 6, 15–29. doi: 10.1023/A:1009524612420

[ref97] Paa H. K. Larson L. M. (1998). Predicting level of restrained eating behavior in adult women. Int. J. Eat. Disord. 24, 91–94. doi: 10.1002/(SICI)1098-108X(199807)24:1<91::AID-EAT8>3.0.CO;2-W, PMID: 9589314

[ref98] Palve S. S. Palve S. B. (2018). Impact of aging on nerve conduction velocities and late responses in healthy individuals. J. Neurosci. Rural Pract. 9, 112–116. doi: 10.4103/jnrp.jnrp_323_17, PMID: 29456354 PMC5812134

[ref99] Paradise A. W. Kernis M. H. (2002). Self-esteem and psychological well-being: implications of fragile self-esteem. J. Soc. Clin. Psychol. 21, 345–361. doi: 10.1521/jscp.21.4.345.22598

[ref100] Parsons L. M. (1987). Imagined spatial transformation of one’s body. J. Exp. Psychol. Gen. 116, 172–191. doi: 10.1037/0096-3445.116.2.172, PMID: 2955072

[ref102] Peirce J. (2009). Generating stimuli for neuroscience using PsychoPy. Front. Neuroinform. 2:2008. doi: 10.3389/neuro.11.010.2008, PMID: 19198666 PMC2636899

[ref1004] Perceiving Systems MPI IS (2011). Body Visualizer. MPI IS Perceiving Systems Department, Copyright Max Planck Gesellschaft. Available at: http://bodyvisualizer.com

[ref103] Verdú E. Ceballos D. Vilches J. J. Navarro X. (2000). Influence of aging on Peripheral nerve function and regeneration. J. Peripher Nerv. Syst. 5, 191–208. doi: 10.1046/j.1529-8027.2000.00026.x 11151980

[ref104] Prnjak K. Jukic I. Mitchison D. Griffiths S. Hay P. (2022). Body image as a multidimensional concept: a systematic review of body image facets in eating disorders and muscle dysmorphia. Body Image 42, 347–360. doi: 10.1016/j.bodyim.2022.07.006, PMID: 35926364

[ref105] Probst M. Pieters G. Vanderlinden J. (2008). Evaluation of body experience questionnaires in eating disorders in female patients (AN/BN) and nonclinical participants. Int. J. Eat. Disord. 41, 657–665. doi: 10.1002/eat.20531, PMID: 18446834

[ref106] Qu F. Wang J. Zhong Y. Ye H. (2018). Postural effects on the mental rotation of body-related pictures: an fMRI study. Front. Psychol. 9:720. doi: 10.3389/fpsyg.2018.00720, PMID: 29875713 PMC5975102

[ref107] Quittkat H. L. Hartmann A. S. Düsing R. Buhlmann U. Vocks S. (2019). Body dissatisfaction, importance of appearance, and body appreciation in men and women over the lifespan. Front. Psych. 10:864. doi: 10.3389/fpsyt.2019.00864, PMID: 31920737 PMC6928134

[ref108] R Core Team. (2021). R: A language and environment for statistical computing. R foundation for statistical computing. Available at: https://www.R-project.org/

[ref109] Raimo S. Di Vita A. Boccia M. Iona T. Cropano M. Gaita M.. (2021). The body across the lifespan: on the relation between interoceptive sensibility and high-order body representations. Brain Sci. 11:493. doi: 10.3390/brainsci11040493 33924634 PMC8070580

[ref111] Reboussin B. A. Rejeski W. J. Martin K. A. Callahan K. Dunn A. L. King A. C.. (2000). Correlates of satisfaction with body function and body appearance in middle- and older aged adults: the activity counseling trial (ACT). Psychol. Health 15, 239–254. doi: 10.1080/08870440008400304

[ref112] Riva G. (2014). Out of my real body: cognitive neuroscience meets eating disorders. Front. Hum. Neurosci. 8:236. doi: 10.3389/fnhum.2014.00236, PMID: 24834042 PMC4018545

[ref113] Riva G. Dakanalis A. (2018). Altered processing and integration of multisensory bodily representations and signals in eating disorders: a possible path toward the understanding of their underlying causes. Front. Hum. Neurosci. 12:49. doi: 10.3389/fnhum.2018.00049 29483865 PMC5816057

[ref114] Robins R. Trzesniewski K. (2005). Self-esteem development across the lifespan. Curr. Direct. Psychol. Sci. 14, 158–162. doi: 10.1111/j.0963-7214.2005.00353.x

[ref115] Rodgers R. F. Laveway K. Campos P. Carvalho P. H. B. de. (2023). Body image as a global mental health concern. Cambridge Prisms, 10,:e9. doi: 10.1017/gmh.2023.2, PMID: 36861019 PMC9970735

[ref116] Roy M. Payette H. (2012). The body image construct among Western seniors: a systematic review of the literature. Arch. Gerontol. Geriatr. 55, 505–521. doi: 10.1016/j.archger.2012.04.007, PMID: 22578668

[ref117] Rudiger J. A. Cash T. F. Roehrig M. Thompson J. K. (2007). Day-to-day body-image states: prospective predictors of intra-individual level and variability. Body Image 4, 1–9. doi: 10.1016/j.bodyim.2006.11.004, PMID: 18089247

[ref118] Runfola C. D. Von Holle A. Trace S. E. Brownley K. A. Hofmeier S. M. Gagne D. A.. (2013). Body dissatisfaction in women across the lifespan: results of the UNC-SELF and gender and body image (GABI) studies. Eur. Eat. Disord. Rev. 21, 52–59. doi: 10.1002/erv.2201, PMID: 22949165 PMC3745223

[ref119] Samuels K. Maine M. Tantillo M. (2019). Disordered eating, eating disorders, and body image in midlife and older women. Curr. Psychiatry Rep. 21:70. doi: 10.1007/s11920-019-1057-5 31264039

[ref120] Sánchez-Cabrero R. Martínez-López F. Euán-Ramírez R. G. (2020). Body image of people over 50 in Spain measured using the BSQ test. BMC. Res. Notes 13:50. doi: 10.1186/s13104-020-4913-9, PMID: 32000851 PMC6993371

[ref121] Schwoebel J. Friedman R. Duda N. Coslett H. B. (2001). Pain and the body schema: evidence for peripheral effects on mental representations of movement. Brain: a. J. Neurol. 124, 2098–2104. doi: 10.1093/brain/124.10.2098 11571225

[ref122] Serino S. Dakanalis A. Gaudio S. Carrà G. Cipresso P. Clerici M.. (2015). Out of body, out of space: impaired reference frame processing in eating disorders. Psychiatry Res. 230, 732–734. doi: 10.1016/j.psychres.2015.10.025, PMID: 26541204

[ref123] Sherrington C. S. (1906). The integrative action of the nervous system.Yale, USA: Yale University Press. doi: 10.1037/13798-000

[ref124] Sirigu A. Duhamel J. R. Cohen L. Pillon B. Dubois B. Agid Y. (1996). The mental representation of hand movements after parietal cortex damage. Science (New York, N.Y.), 273, 1564–1568. doi: 10.1126/science.273.5281.1564 8703221

[ref125] Skrzypek S. Wehmeier P. M. Remschmidt H. (2001). Body image assessment using body size estimation in recent studies on anorexia nervosa. A brief review. Eur. Child Adolesc. Psychiatry 10, 215–221. doi: 10.1007/s007870170010, PMID: 11794546

[ref126] Slater A. Tiggemann M. (2015). Media exposure, extracurricular activities, and appearance-related comments as predictors of female adolescents’ self-objectification. Psychol. Women Q. 39, 375–389. doi: 10.1177/0361684314554606

[ref127] Slevec J. H. Tiggemann M. (2011). Predictors of body dissatisfaction and disordered eating in middle-aged women. Clin. Psychol. Rev. 31, 515–524. doi: 10.1016/j.cpr.2010.12.002 21239098

[ref128] Soares C. N. Zitek B. (2008). Reproductive hormone sensitivity and risk for depression across the female life cycle: a continuum of vulnerability? J. Psychiatry. Neurosci. 33, 331–343. PMID: 18592034 PMC2440795

[ref129] St-Onge M.-P. Gallagher D. (2010). Body composition changes with aging: the cause or the result of alterations in metabolic rate and macronutrient oxidation? Nutrition 26, 152–155. doi: 10.1016/j.nut.2009.07.004, PMID: 20004080 PMC2880224

[ref130] Stice E. Shaw H. E. (2002). Role of body dissatisfaction in the onset and maintenance of eating pathology: a synthesis of research findings. J. Psychosom. Res. 53, 985–993. doi: 10.1016/S0022-3999(02)00488-9, PMID: 12445588

[ref131] Thakkar K. N. Brugger P. Park S. (2009). Exploring empathic space: correlates of perspective transformation ability and biases in spatial attention. PLoS One 4:e5864. doi: 10.1371/journal.pone.0005864, PMID: 19516894 PMC2688758

[ref132] Thornborrow T. Evans E. H. Tovee M. J. Boothroyd L. G. (2022). Sociocultural drivers of body image and eating disorder risk in rural Nicaraguan women. J. Eat. Disord. 10:133. doi: 10.1186/s40337-022-00656-0, PMID: 36068623 PMC9450464

[ref133] Tiggemann M. (2004). Body image across the adult life span: stability and change. Body Image 1, 29–41. doi: 10.1016/S1740-1445(03)00002-0, PMID: 18089139

[ref134] Tiggemann M. Lynch J. E. (2001). Body image across the life span in adult women: the role of self-objectification. Dev. Psychol. 37, 243–253. doi: 10.1037/0012-1649.37.2.243, PMID: 11269392

[ref135] Tiggemann M. McCourt A. (2013). Body appreciation in adult women: relationships with age and body satisfaction. Body Image 10, 624–627. doi: 10.1016/j.bodyim.2013.07.003, PMID: 23954196

[ref136] Todd J. Aspell J. E. Barron D. Swami V. (2019a). An exploration of the associations between facets of interoceptive awareness and body image in adolescents. Body Image 31, 171–180. doi: 10.1016/j.bodyim.2019.10.004, PMID: 31654981

[ref137] Todd J. Aspell J. E. Barron D. Swami V. (2019b). Multiple dimensions of interoceptive awareness are associated with facets of body image in British adults. Body Image 29, 6–16. doi: 10.1016/j.bodyim.2019.02.003, PMID: 30771695

[ref139] Tsakiris M. Jiménez A. T. Costantini M. (2011). Just a heartbeat away from one's body: interoceptive sensitivity predicts malleability of body-representations. Proc. R. Soc. B Biol. Sci. 278, 2470–2476. doi: 10.1098/rspb.2010.2547, PMID: 21208964 PMC3125630

[ref140] Tylka T. L. (2004). The relation between body dissatisfaction and eating disorder symptomatology: an analysis of moderating variables. J. Couns. Psychol. 51, 178–191. doi: 10.1037/0022-0167.51.2.178

[ref1003] United Nations, Department of Economic and Social Affairs, Population Division. (2015). World Population Ageing 2015 (ST/ESA/SER.A/390).

[ref141] United Nations. (2019). United Nations development Programme, human development report. 2019. ‘Human development index.’ Available at: http://hdr.undp.org/en/composite/HDI.

[ref142] Urgesi C. Fornasari L. De Faccio S. Perini L. Mattiussi E. Ciano R.. (2011). Body schema and self-representation in patients with bulimia nervosa. Int. J. Eat. Disord. 44, 238–248. doi: 10.1002/eat.20816, PMID: 20186715

[ref143] Vaitl D. (1996). Interoception. Biol. Psychol. 42, 1–27. doi: 10.1016/0301-0511(95)05144-9 8770368

[ref144] Wade T. (2016). “Body shape questionnaire” in Encyclopedia of feeding and eating disorders. ed. Wade T. (Singapore: Springer), 96–99. doi: 10.1007/978-981-287-087-2_212-1, PMID:

[ref145] Wang S. B. Haynos A. F. Wall M. M. Chen C. Eisenberg M. E. Neumark-Sztainer D. (2019). Fifteen-year prevalence, trajectories, and predictors of body dissatisfaction from adolescence to middle adulthood. Clin. Psychol. Sci. 7, 1403–1415. doi: 10.1177/2167702619859331, PMID: 32864198 PMC7451946

[ref146] Wignall S. J. Thomas N. A. Nicholls M. E. R. (2017). Fat or fiction? Effects of body size, eating pathology, and sex upon the body schema of an undergraduate population. Body Image 23, 135–145. doi: 10.1016/j.bodyim.2017.09.004 28992582

[ref147] Zacks J. M. Mires J. Tversky B. Hazeltine E. (2000). Mental spatial transformations of objects and perspective. Spat. Cogn. Comput. 2, 315–332. doi: 10.1023/A:1015584100204

[ref148] Zacks J. Rypma B. Gabrieli J. D. E. Tversky B. Glover G. H. (1999). Imagined transformations of bodies: an fMRI investigation. Neuropsychologia 37, 1029–1040. doi: 10.1016/S0028-3932(99)00012-3, PMID: 10468366

