# Supplementary material for: Body image disturbance, interoceptive sensibility and the body schema across female adulthood: a pre-registered study
Source: Front Psychol. 2023 Nov 30;14:1285216. doi: 10.3389/fpsyg.2023.1285216 (PMC10720753; doi:10.3389/fpsyg.2023.1285216)
Supplement: Supplementary file 1 [file Data_Sheet_1.docx]

**Supplementary Tables and Figures**

| S1. Regression coefficients (Standardised and Unstandardised) for the regression model, IA, Age entered as predictors and BID composite score as dependant variable. | | | | | | | | | | | | | |
| --- | --- | --- | --- | --- | --- | --- | --- | --- | --- | --- | --- | --- | --- |
| **Model** | |  | | **Unstandardized** | | **Standard Error** | | **Standardized** | | **t** | | **p** | |
| H₀ |  | (Intercept) |  | 0.225 |  | 0.157 |  |  |  | 1.431 |  | 0.153 |  |
|  |  | Adults |  | -0.005 |  | 0.222 |  |  |  | -0.022 |  | 0.982 |  |
|  |  | Middle Adults |  | -0.117 |  | 0.223 |  |  |  | -0.527 |  | 0.598 |  |
|  |  | Older Adults |  | -0.758 |  | 0.220 |  |  |  | -3.449 |  | < .001 |  |
| H₁ |  | (Intercept) |  | 0.266 |  | 0.150 |  |  |  | 1.769 |  | 0.077 |  |
|  |  | Adults |  | 0.064 |  | 0.213 |  |  |  | 0.299 |  | 0.765 |  |
|  |  | Middle Adults |  | -0.175 |  | 0.213 |  |  |  | -0.821 |  | 0.412 |  |
|  |  | Older Adults |  | -0.928 |  | 0.211 |  |  |  | -4.406 |  | < .001 |  |
|  |  | Interoceptive Sensibility |  | -0.805 |  | 0.075 |  | -0.294 |  | -10.700 |  | < .001 |  |
|  | | | | | | | | | | | | | |
|  | | | | | | | | | | | | | |

| S2. Pearson’s partial correlations of all the questionnaires, conditioned on age group. | | | | | | | | | | | | |
| --- | --- | --- | --- | --- | --- | --- | --- | --- | --- | --- | --- | --- |
| **Questionnaire** | |  | | **BSQ** | | **BISS** | | **Body Surveillance** | | **Body Shame** | | **MAIA-2** |
| BSQ |  | Pearson's r |  | — |  |  |  |  |  |  |  |  |
|  |  | p-value |  | — |  |  |  |  |  |  |  |  |
| BISS |  | Pearson's r |  | 0.733 | *** | — |  |  |  |  |  |  |
|  |  | p-value |  | < .001 |  | — |  |  |  |  |  |  |
| Body Surveillance |  | Pearson's r |  | 0.588 | *** | 0.475 | *** | — |  |  |  |  |
|  |  | p-value |  | < .001 |  | < .001 |  | — |  |  |  |  |
| Body Shame |  | Pearson's r |  | 0.757 | *** | 0.622 | *** | 0.625 | *** | — |  |  |
|  |  | p-value |  | < .001 |  | < .001 |  | < .001 |  | — |  |  |
| MAIA-2 |  | Pearson's r |  | -0.200 | *** | -0.310 | *** | -0.249 | *** | -0.183 | *** | — |
|  |  | p-value |  | < .001 |  | < .001 |  | < .001 |  | < .001 |  | — |
|  | | | | | | | | | | | | |
| * p < .05, ** p < .01, *** p < .001 | | | | | | | | | | | | |

| S.3 Full reported ANOVA post-hoc results investigating interactions between Condition (control, experimental) and Position (front-facing, back-facing). | | | | | | | | | | | | | |
| --- | --- | --- | --- | --- | --- | --- | --- | --- | --- | --- | --- | --- | --- |
|  | |  | | **Mean Difference** | | **SE** | | **t** | | **Cohen's d** | | **p_bonf_** | |
| Back-facing Control |  | Front-facing Control |  | 7.145 |  | 0.570 |  | 12.529 |  | 0.510 |  | < .001 |  |
|  |  | Back-facing Experimental |  | 1.220 |  | 0.568 |  | 2.148 |  | 0.087 |  | 0.191 |  |
|  |  | Front-facing Experimental |  | 7.923 |  | 0.570 |  | 13.898 |  | 0.565 |  | < .001 |  |
| Front-facing Control |  | Back-facing Experimental |  | -5.926 |  | 0.571 |  | -10.386 |  | -0.423 |  | < .001 |  |
|  |  | Front-facing Experimental |  | 0.778 |  | 0.573 |  | 1.358 |  | 0.055 |  | 1.000 |  |
| Back-facing Experimental |  | Front-facing Experimental |  | 6.704 |  | 0.570 |  | 11.754 |  | 0.478 |  | < .001 |  |
|  | | | | | | | | | | | | | |
| Note.  P-value adjusted for comparing a family of 4 | | | | | | | | | | | | | |

| S.4 Full reported ANOVA results of 2x2x2x4 Omnibus Mixed-Model ANOVA Design with Posture (front-facing, back-facing), Orientation (0 degrees, 90 degrees), Weight (underweight, overweight), and Age group) as Between-Subjects Factors. | | | | | | | | | | | | | |
| --- | --- | --- | --- | --- | --- | --- | --- | --- | --- | --- | --- | --- | --- |
|  | | **Sum of Squares** | | **df** | | **Mean Square** | | **F** | | **p** | | **η²_p_** | |
| Position |  | 258.906 |  | 1 |  | 258.906 |  | 8.6831 |  | 0.003 |  | 0.007 |  |
| Position ✻ Age group |  | 115.298 |  | 3 |  | 38.433 |  | 1.2889 |  | 0.277 |  | 0.003 |  |
| Residual |  | 37629.524 |  | 1262 |  | 29.817 |  |  |  |  |  |  |  |
| Orientation |  | 0.339 |  | 1 |  | 0.339 |  | 0.0106 |  | 0.918 |  | 0.000 |  |
| Orientation ✻ Age group |  | 270.174 |  | 3 |  | 90.058 |  | 2.8009 |  | 0.039 |  | 0.007 |  |
| Residual |  | 40577.329 |  | 1262 |  | 32.153 |  |  |  |  |  |  |  |
| Weight |  | 11.872 |  | 1 |  | 11.872 |  | 0.3998 |  | 0.527 |  | 0.000 |  |
| Weight ✻ Age group |  | 90.943 |  | 3 |  | 30.314 |  | 1.0209 |  | 0.382 |  | 0.002 |  |
| Residual |  | 37474.515 |  | 1262 |  | 29.695 |  |  |  |  |  |  |  |
| Position ✻ Orientation |  | 13.187 |  | 1 |  | 13.187 |  | 0.4437 |  | 0.505 |  | 0.000 |  |
| Position ✻ Orientation ✻ Age group |  | 101.822 |  | 3 |  | 33.941 |  | 1.1421 |  | 0.331 |  | 0.003 |  |
| Residual |  | 37505.030 |  | 1262 |  | 29.719 |  |  |  |  |  |  |  |
| Position ✻ Weight |  | 16.517 |  | 1 |  | 16.517 |  | 0.5607 |  | 0.454 |  | 0.000 |  |
| Position ✻ Weight ✻ Age_group |  | 80.086 |  | 3 |  | 26.695 |  | 0.9062 |  | 0.437 |  | 0.002 |  |
| Residual |  | 37178.272 |  | 1262 |  | 29.460 |  |  |  |  |  |  |  |
| Orientation ✻ Weight |  | 34.526 |  | 1 |  | 34.526 |  | 1.1651 |  | 0.281 |  | 0.001 |  |
| Orientation ✻ Weight ✻ Age_group |  | 73.697 |  | 3 |  | 24.566 |  | 0.8290 |  | 0.478 |  | 0.002 |  |
| Residual |  | 37396.769 |  | 1262 |  | 29.633 |  |  |  |  |  |  |  |
| Position ✻ Orientation ✻ Weight |  | 37.370 |  | 1 |  | 37.370 |  | 1.2629 |  | 0.261 |  | 0.001 |  |
| Position ✻ Orientation ✻ Weight ✻ Age_group |  | 82.639 |  | 3 |  | 27.546 |  | 0.9309 |  | 0.425 |  | 0.002 |  |
| Residual |  | 37344.359 |  | 1262 |  | 29.591 |  |  |  |  |  |  |  |
| Note. Type 3 Sums of Squares | | | | | | | | | | | | | |
|  | | | | | | | | | | | | | |

| S.5. Regression coefficients (Standardised and Unstandardised) for the regression model, BID, IA, Age and Orientation entered as predictors and egocentric transformation cost entered as outcome. | | | | | | | | | | | | | | |
| --- | --- | --- | --- | --- | --- | --- | --- | --- | --- | --- | --- | --- | --- | --- |
| **Model** | |  | | **Unstandardized**  **coefficient** | | **Standard Error** | | **Standardized coefficient** | | **t** | | | **p** | |
| H₀ |  | (Intercept) |  | 504.540 |  | 16.994 |  |  |  | 29.689 |  | < .001 | |  |
|  |  | Adults |  | -8.824 |  | 21.569 |  |  |  | -0.409 |  | 0.682 | |  |
|  |  | Middle Adults |  | -35.129 |  | 21.541 |  |  |  | -1.631 |  | 0.103 | |  |
|  |  | Older Adults |  | -63.899 |  | 21.362 |  |  |  | -2.991 |  | 0.003 | |  |
|  |  | orientation (90) |  | -237.188 |  | 15.203 |  |  |  | -15.602 |  | < .001 | |  |
| H₁ |  | (Intercept) |  | 502.647 |  | 17.002 |  |  |  | 29.564 |  | < .001 | |  |
|  |  | Body Image Disturbance |  | 7.425 |  | 2.931 |  | 0.052 |  | 2.534 |  | 0.011 | |  |
|  |  | Adults |  | -9.209 |  | 21.558 |  |  |  | -0.427 |  | 0.669 | |  |
|  |  | Middle Adults |  | -34.107 |  | 21.534 |  |  |  | -1.584 |  | 0.113 | |  |
|  |  | Older Adults |  | -57.937 |  | 21.572 |  |  |  | -2.686 |  | 0.007 | |  |
|  |  | Orientation (90) |  | -237.182 |  | 15.188 |  |  |  | -15.616 |  | < .001 | |  |
|  |  | Interoceptive awareness |  | 3.174 |  | 8.021 |  | 0.008 |  | 0.396 |  | 0.692 | |  |
|  | | | | | | | | | | | | | | |

S.6. Pearson's Partial Correlations of all the questionnaires and transformations Frontfacing and Backfacing Reaction time scores in 0 degrees and 90 degrees, conditioned on age group.

|  | |  | | BACKFACING 0 | | BACKFACING 90 | | FRONTFACING 0 | | FRONTFACING 90 | |
| --- | --- | --- | --- | --- | --- | --- | --- | --- | --- | --- | --- |
| Body Shape Questionnaire |  | Pearson's r |  | -0.004 |  | 0.036 |  | 0.051 |  | 0.095 | *** |
|  |  | p-value |  | 0.891 |  | 0.209 |  | 0.077 |  | < .001 |  |
| Body Image State scale |  | Pearson's r |  | 0.001 |  | 0.023 |  | 0.048 |  | 0.083 | ** |
|  |  | p-value |  | 0.965 |  | 0.432 |  | 0.096 |  | 0.004 |  |
| Body Surveillance subscale |  | Pearson's r |  | 0.018 |  | -0.007 |  | 0.013 |  | 0.054 |  |
|  |  | p-value |  | 0.530 |  | 0.821 |  | 0.648 |  | 0.062 |  |
| Body Shame Subscale |  | Pearson's r |  | 0.019 |  | 0.059 | * | 0.041 |  | 0.082 | ** |
|  |  | p-value |  | 0.501 |  | 0.041 |  | 0.154 |  | 0.004 |  |
| Interoceptive awareness (MAIA-2) |  | Pearson's r |  | -0.018 |  | 0.015 |  | 0.055 |  | 0.055 |  |
|  |  | p-value |  | 0.532 |  | 0.599 |  | 0.056 |  | 0.057 |  |

Significance as follows: * p < .05, ** p < .01, *** p < .001


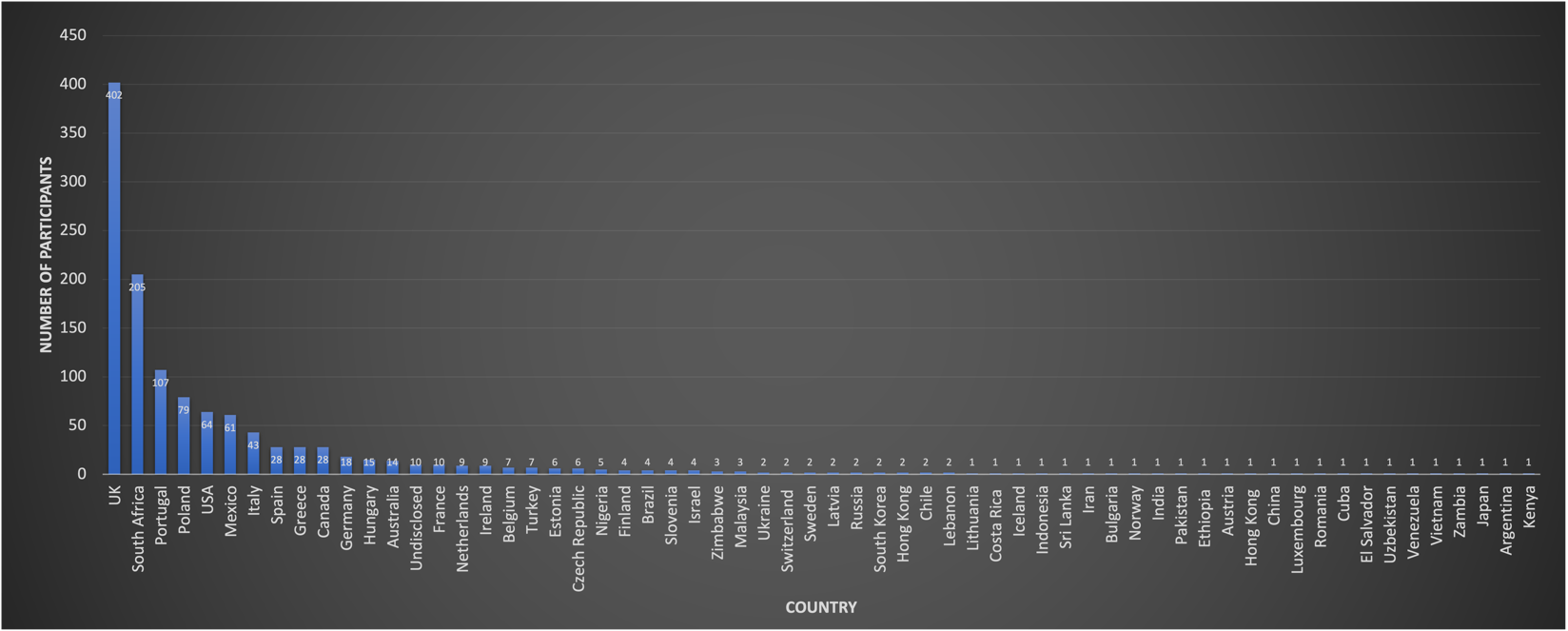


S.7 Breakdown of number of participants by country

S.8 For the age related difference in MAIA-2 scores, the new index also revealed a significant difference between the 4 age groups (F(3,1210) = 2.976, p = 0.031, η^2^_p_ = 0.007). Post hoc comparisons indicated there were higher IS scores in the Adult group compared to the Older Adult group (*t* = 2.880, *SE* = 0.037,*p* = 0.024, *d* = 0.235).
